# Mutations in the Transcription Elongation Factor SPT5 Disrupt a Reporter for Dosage Compensation in Drosophila

**DOI:** 10.1371/journal.pgen.1003073

**Published:** 2012-11-29

**Authors:** Mahalakshmi Prabhakaran, Richard L. Kelley

**Affiliations:** 1 Program in Developmental Biology, Baylor College of Medicine, Houston, Texas, United States of America; 2 Department of Molecular and Human Genetics, Baylor College of Medicine, Houston, Texas, United States of America; Wayne State University, United States of America

## Abstract

In *Drosophila*, the MSL (Male Specific Lethal) complex up regulates transcription of active genes on the single male X-chromosome to equalize gene expression between sexes. One model argues that the MSL complex acts upon the elongation step of transcription rather than initiation. In an unbiased forward genetic screen for new factors required for dosage compensation, we found that mutations in the universally conserved transcription elongation factor *Spt5* lower MSL complex dependent expression from the *miniwhite* reporter gene *in vivo*. We show that SPT5 interacts directly with MSL1 *in vitro* and is required downstream of MSL complex recruitment, providing the first mechanistic data corroborating the elongation model of dosage compensation.

## Author Summary

Drosophila males hypertranscribe most of the genes along their single X chromosome to match the output of females with two X chromosomes. It had been difficult to imagine how the MSL dosage compensation complex could impose a modest, but essential, ∼two-fold increase by interacting with hundreds of different factors that control transcription initiation for such a diverse collection of genes. An alternative model proposed that dosage compensation instead acted at some step of transcription elongation common to all genes. We performed a genetic screen for mutations that subtly reduce dosage compensation and recovered mutations in the *Spt5* gene that encodes a universally conserved elongation factor. SPT5 closes the RNA polymerase II clamp around the DNA template to prevent pausing or premature termination. We find that the dosage compensation complex genetically and physically interacts with SPT5 on actively transcribed genes providing direct molecular support for the elongation model of dosage compensation.

## Introduction

*Drosophila* dosage compensation is widely used as a model system to investigate how transcription is regulated by large scale chromatin modifications [1]. To equalize the expression of the X-linked genes between XY males and XX females, the single X-chromosome in males is hypertranscribed a modest, but essential ∼1.4–1.8 fold. This is accomplished by the MSL complex, which consists of at least five proteins and two noncoding *roX* (RNA on X) RNAs [2]. The complex contains the histone modifying enzymes MOF (H4K16ac) and MSL2 (H2BK34ub) [3]. MSL3 is a chromodomain protein implicated in MSL complex distribution to its target site [4]. MSL1 assembles the complex via discrete docking sites for MSL2, MSL3, and MOF. MLE is an ATPase/helicase with double stranded RNA binding motifs that associates with the complex in an RNA dependent manner.

A long-standing puzzle is the biochemical mechanism by which the MSL complex up regulates X-linked genes, each of which is controlled by different transcription factors. An elegant model that solves this problem posits that MSL complex does not act with diverse gene-specific transcription factors to alter initiation, but rather at the elongation step of transcription common to all genes [5]. This proposal is supported by the higher resolution mapping of MSL complex binding and H4K16 acetylation within the bodies of actively transcribed X-linked genes with a bias towards the 3′ end [6]–[8]. Global nuclear run on analysis showed that compared to autosomes, the male X-chromosome has higher levels of transcriptionally engaged RNAPII (RNA Polymerase II) within the distal portions of the genes [9]. In contrast to this, a recent study detected increased RNAPII occupancy at the promoters of X-linked genes in males leading to the alternate idea that dosage compensation operates at the level of transcription initiation [10]. It is not clear whether decondensation of the chromatin fiber by H4K16 acetylation aids passage or recruitment of RNAPII enough to explain dosage compensation [11], or if the MSL complex has additional interactions with the basal transcriptional machinery.

To search for new factors involved in dosage compensation we performed an unbiased forward genetic screen that relies on a sensitive eye pigmentation reporter of MSL complex activity. This approach was designed to recover heterozygous mutations in genes that are essential for general transcription in both sexes but play an additional role in male dosage compensation. We recovered multiple alleles of *Spt5*, a universally conserved transcription elongation factor. We found that SPT5 is required for dosage compensation in males and extensively colocalizes with the MSL complex on the X-chromosome. Moreover, we found that SPT5 and MSL1 directly interact with each other. We propose that SPT5 is required downstream of MSL complex recruitment to stimulate transcription elongation. The identification of SPT5 is strong mechanistic evidence supporting the elongation model of dosage compensation.

## Results

### A genetic screen for identifying new components of dosage compensation

The eye color of *roX1* transgenic males is a sensitive reporter of MSL activity [12]. When *roX1* transgenes occasionally land in repressive chromatin, the *miniwhite* marker is epigenetically silenced so that females have solid white eyes. Males have spotted eyes because the MSL complex binds the autosomal *roX1* transgene and locally modifies the chromatin allowing *miniwhite* expression in a fraction of cells. We have previously described a strategy for isolating mutations that increased local MSL activity [13]. Here we use a similar method to isolate mutations that reduce MSL activity. This approach has two important advantages. First, it allows identification of factors that are instrumental in achieving dosage compensation of the male X-chromosome *in vivo*, but may associate with the MSL complex too weakly or transiently to copurify with MSL proteins. Second, our genetic strategy retains a wild type allele of the relevant gene allowing us to capture factors that have additional essential functions. Homozygous mutations in such factors would be lethal to both sexes and thus would have been missed in earlier genetic screens based on the male specific lethal phenotype.

**Figure 1:**
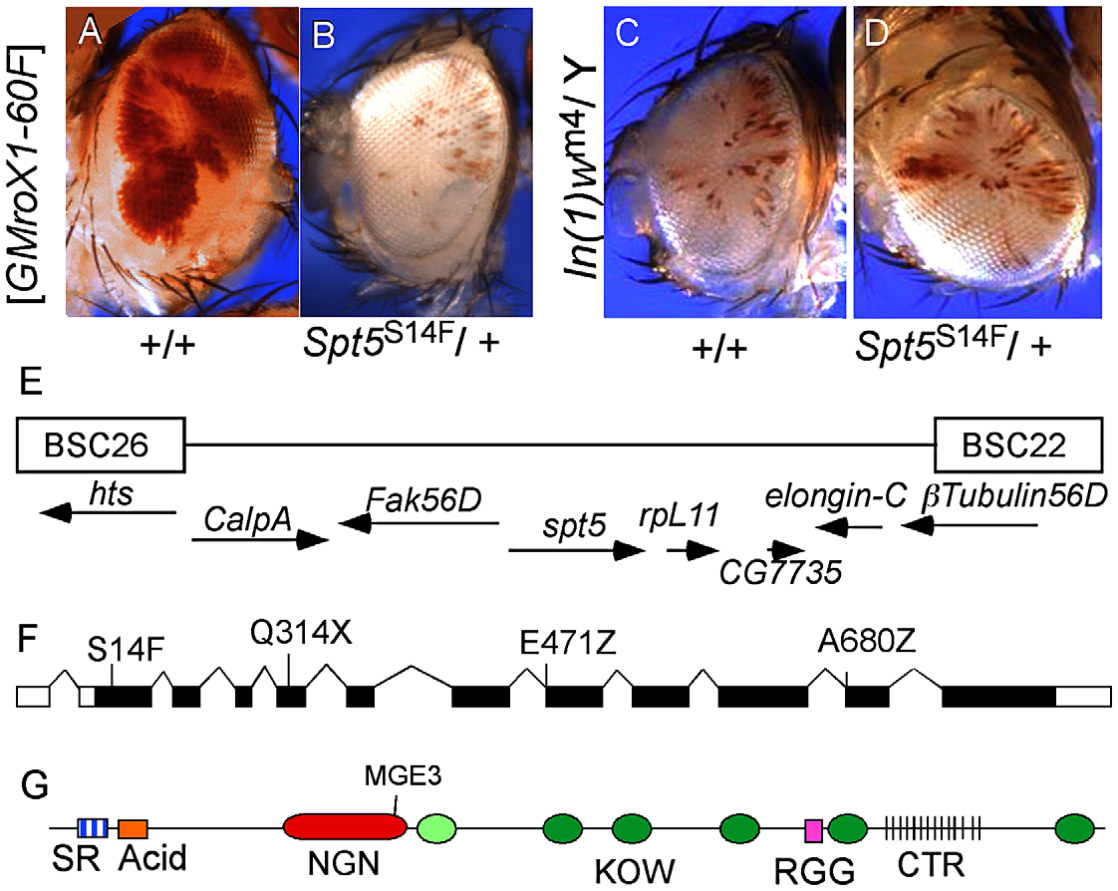
Isolation of *Spt5* mutations. (A) Male flies carrying the *[w^+^ GMroX1]* transgene inserted at the 2R telomere (60F) have sectored pigmentation due to dosage compensation at the transgene. (B) Males heterozygous for mutations in *Spt5* lose most red eye pigmentation due to reduced MSL complex activity. (C and D) *Spt5* mutants have no effect on the PEV line *In(1)w^m4^*. (E) Genomic location of *Spt5* and flanking deficiencies. (F) New *Spt5* mutations failed to complement the previously reported *Spt5^MGE-3^* allele [15]. (G) SPT5 domain features. SR, Serine/Arginine; NGN, N-terminal NusG; KOW, Kyrpides, Ouzounis, Woese light green oval indicates only partial match to consensus; RGG, arginine glycine glycine repeats; CTR, C-Terminal Repeats similar to RNAPII. Screen design and results are in Figure S1.

We screened approximately 16,000 EMS mutagenized flies and identified 48 mutations that dramatically lowered the eye pigmentation in males (Figure 1A and 1B, Figure S1). It is difficult to estimate if the amount of pigmentation in any individual ommatidia changes. What the screen detects is a change in the fraction of ommatidia that do or do not derepress the *miniwhite* reporter linked to *roX1*. Mutants that were also recessive lethal to both sexes were placed in complementation groups (Figure S1). We tested the ability of the modifier mutants to suppress eye pigmentation in multiple mosaic *roX1* transgenes inserted in distinct repressive locations reasoning that those were more likely to affect dosage compensation rather than the particular silencing factors acting on flanking chromatin (Figure S2). One uninteresting mechanism that might produce this phenotype would be mutations that globally strengthened the repressive chromatin environment responsible for silencing of the *miniwhite* gene in our *roX1* reporters. We tested the effect of the new mutants on *In(1)w^m4^* which displays classic position effect variegation in both sexes. Most of the candidate modifiers of dosage compensation did not affect pigmentation in *In(1)w^m4^* (Figure 1C and 1D) arguing against a global increase in repressive chromatin.

### SPT5 couples dosage compensation and transcription elongation

Complementation group C was chosen for detailed analysis. Meiotic recombination placed the locus near the polytene bands 56C-F but none of the available chromosome deficiencies uncovered the mutation [14]. Closer inspection revealed a gap in the deficiencies where elongation factors *Spt5* and *Elongin-C* are located (Figure 1E). Available mutations in *Elongin-C* complemented all five group C alleles and we found no lesions in *Elongin-C* upon sequencing (data not shown). However, when we tested the *Spt5^MGE-3^* mutation [15], it failed to complement all five group C alleles. Sequencing genomic DNA from these mutants identified one stop codon (Q314X), two splice junction mutations (E471Z and A680Z), and one missense mutation (S14F) (Figure 1F). The modular structure of SPT5 is summarized in Figure 1G.

Because SPT5 is such a critically important transcription elongation factor used by many genes, we were concerned that it appeared in our screen because reducing the level of any vital general transcription factor would lower expression of our eye color reporter. To address this concern, we screened the autosomal Bloomington Deficiency stock collection. We reasoned that if disrupting transcriptional efficiency in general affected our reporter, then many deficiencies would lower red pigmentation of the mosaic *roX1* lines just like *Spt5* mutations had. We crossed six different *roX1* mosaic lines that carry *roX1* transgenes in diverse chromatin environments to 190 deficiencies. The idea was that any deficiency that affected the eye coloration of multiple *roX1* reporter lines was more likely to affect some aspect of dosage compensation rather than the particular repressive environment surrounding the different inserts. We found that only 10 intervals reduced MSL complex reporter activity (Table S1). Moreover, removing one copy of these 10 regions lowered MSL complex dependent red pigmentation across 4 or more of the mosaic *roX1* lines supporting the notion that the relevant factors are somehow acting on dosage compensation. The deficiency screen shows that silencing the *roX1* eye color reporter is an uncommon dominant haploinsufficient phenotype produced by only a few loci in the genome. Thus, the phenotype seen in *Spt5* mutants is unlikely to be due to a general reduction of transcription.

We were still concerned that the *white* eye color gene used in our dosage compensation reporter might be particularly sensitive to SPT5 levels. We turned to strong hypomorphic alleles of *white* to test this possibility. The *w* ^a^ and *w* ^e^ alleles each carry different transposon insertions that greatly reduce their expression resulting in orange eyes [17], [18]. On this background, small changes in *white* expression should be easily detectable by altered eye color. We crossed our *Spt5* mutations into these two stocks and observed no difference in the eye pigmentation (Figure S3). We also crossed unrelated transgenes marked with *miniwhite* into our new *Spt5* mutations and saw no change in eye pigmentation (data not shown). This shows that a 50% reduction in SPT5 levels does not alter the phenotype from hypomorphic *white* alleles or *miniwhite*. We conclude that *Spt5* mutations dramatically affected the probability that males overcome silencing not because of global reduction of transcription across the genome or the *white* promoter itself, but rather because SPT5 plays some role in dosage compensation to which our *roX1* reporter is responsive.

**Figure 2:**
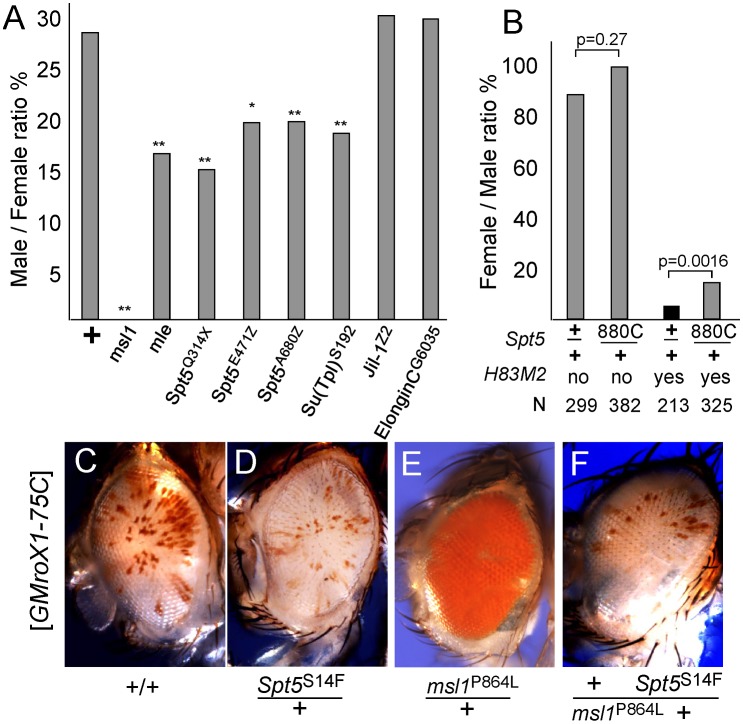
Genetic interactions between *Spt5* and dosage compensation. (A) Lowering SPT5 reduces male viability compared to sisters. All males were *roX1 roX2* double mutants and partially rescued by one copy of the *[GMroX1-75C]* transgene. Males wild type for all other loci are rescued 29%, but flies missing one copy of the indicated dosage compensation genes have reduced male viability. ** p<0.01 * p<0.05 Fisher exact test. Detailed results in Table S2. (B) Reducing *Spt5* rescues the sterility of *[H83M2]* females. *y w; Spt5^880C^/CyO y+* females were mated to *w/Y; [w^+^ H83M2]/+* males. Only the adults eclosing during the first two days are indicated to measure delayed development. *[w^+^ H83M2]* escaper females produced few eggs and were sterile (black bar). [*w^+^ H83M2*] females heterozygous for *Spt5* regained fertility (gray bars). N = number of brothers recovered for each class. p calculated by Fisher exact test. (C) Males homozygous for the *[GMroX1]* transgene at 75C have a few pigmented sectors. (D) Singly, *Spt5* slightly reduces and (E) *msl1^P864L^* dramatically increases local MSL activity. (F) When present together, *Spt5^S14F^* blocks the increased activity of the *msl1^P864L^* gain of function allele. See Figure S2 and Figure S3 for additional genetic analysis.

We used a sensitized genetic background to see if *Spt5* affected dosage compensation of the X chromosome in addition to the *roX1* eye color reporter transgene. Because SPT5 is essential for most transcription, homozygous null animals die early in development. The viability of *Spt5*/+ males demonstrates that dosage compensation must be adequate even with reduced SPT5 levels. The same is true for any of the *msl*/+ heterozygotes. However, males with limiting MSL complex might be more sensitive to reduced levels of SPT5. Males missing either *roX1* or *roX2* are alive but males missing both *roX* RNAs have greatly reduced male viability [19]. In our genetic background such *roX1 roX2* double mutant males are completely lethal but can be rescued by an autosomal *roX* transgene [20]. Restoring male viability under these conditions depends on abundant MSL subunits. Males heterozygous for *msl1* or *mle* showed reduced viability when *roX1* RNA is also limiting [13]. Similarly, reducing SPT5 selectively lowered male viability to ∼15% when *roX1* RNA was limiting consistent with a role in dosage compensation (Figure 2A). We assayed related factors to see how specific this phenotype was and found that lowering *Elongin-C*, another factor involved in elongation or *Jil1*, the histone H3S10 kinase that associates with the MSL complex had no effect on male viability (Figure 2A). However, *Su(Tpl*)^S192^, a mutation in the elongation factor ELL did reduce male viability. Others have reported that *ELL RNAi* lines display male specific lethality consistent with a role in dosage compensation [21]. These results are consistent with SPT5 playing a central role in dosage compensation that becomes more obvious when MSL activity is limited by low *roX1* RNA levels.

Females normally lack dosage compensation because SXL blocks translation of *msl2* mRNA. Ectopic dosage compensation can be induced in females by artificial expression of MSL2 by the [*H83M2*] transgene that escapes SXL regulation [22]. The inappropriate dosage compensation slows development resulting in delayed eclosion of adult females (Figure 2B). The resulting females produce very few eggs and are sterile. If reducing *Spt5* weakens dosage compensation then that might reduce the toxic effects of inappropriate dosage compensation in *[H83M2]* overexpression females. When *[H83M2]* females also carried a mutation in *Spt5*, the female-specific developmental delay was modestly rescued (Figure 2B). However, the more striking result was that the *Spt5*/+ [*H83M2*] females produced abundant eggs that successfully developed into larvae. This argues that SPT5 is needed for MSL2 to drive inappropriate dosage compensation in females.

To further examine functional links between SPT5 and dosage compensation, we tested genetic interactions between the newly recovered *Spt5* mutations and unusual gain of function *msl1* alleles. We previously reported two missense alleles that partially disrupt the MSL1-MOF or MSL1-MSL3 interfaces [13]. Both mutations dominantly cause msl1*/+ males to produce solid red eyes (more MSL activity, Figure 2E) from the mosaic *GMroX1-75C* transgenic reporter whose basal pattern is mostly white with scattered small red sectors (Figure 2C). *Spt5* mutations alone reduce sectoring slightly (Figure 2D). We constructed flies heterozygous for both the *msl1^P864L^* and *Spt5^S14F^* mutations that also carried the 75C dosage compensation eye color reporter. These males had white eyes (Figure 2F). This shows that even MSL complex containing the overly active P864L subunit can only act on the *roX1* reporter when full SPT5 levels are present. Taken together, these *in vivo* results indicate a role for SPT5 in male X- dosage compensation beyond its general role in transcription of the entire genome in both sexes.

### SPT5 and MSL complex colocalize on the X-chromosome

**Figure 3:**
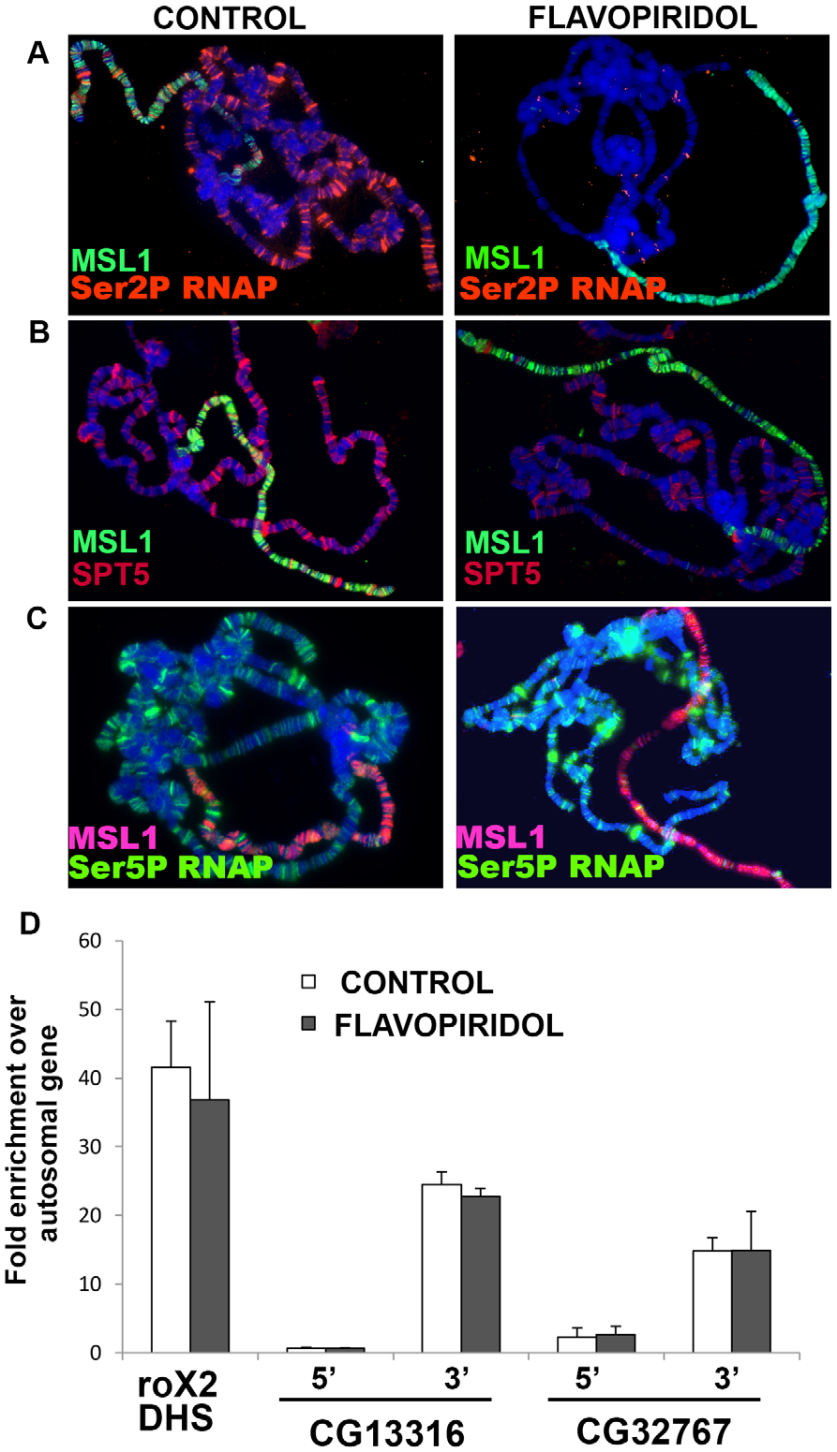
MSL complex binding to X-chromosome is not dependent on actively elongating RNA polymerase. (A) Actively elongating RNA polymerase is found on all chromosomes and largely colocalizes with MSL complex on the male X. Flavopiridol treatment removes elongating polymerase but has no effect on MSL localization. (B) SPT5 decorates all chromosome arms and largely colocalizes with MSL complex on male X. The SPT5 pattern in B is shown more clearly in separate color channels in Figure S5. Flavopiridol treatment removes the bulk of SPT5 from all chromosomes. (C) Flavopiridol treatment has no effect on polymerase paused at the TSS. Chromosomes were stained with indicated antibodies against MSL1, Ser2 phopshorylated RNAP, Ser5 phosphorylated RNAP, and/or SPT5. (D) ChIP analysis of male S2 cells immunoprecipitated with anti-MSL1 antibodies. MSL1 is enriched near the 3′ ends of known MSL1 targets *CG13316* and *CG32767* with comparatively less MSL complex found near the 5′ TSS measured by quantitative real time PCR relative to the autosomal *PKA* gene. Blocking elongation with flavopiridol does not alter the MSL1 distribution. MSL1 binding to the *roX2 DHS* control region occurs by a sequence-dependent mechanism and does not require transcription [62].

ChIP analysis found that SPT5 is enriched over the transcription start site (TSS) of most Drosophila genes, with additional binding across the transcribed regions [23]. At the level of polytene chromosomes, SPT5 binds many sites [16], [24] and colocalizes imperfectly with the MSL complex on the male X-chromosome [6]. To examine this issue in more detail, we raised new SPT5 antibodies. The serum recognized a single band around 135 kDa on SDS-PAGE which is larger than the predicted 119 kDa (Figure S4). Anomalous migration for SPT5 was reported earlier [16], [24]. The SPT5 serum recognized many bands on all polytene chromosomes in both sexes (Figure 3B control panel and Figure S5A). Most of the X-linked bands overlapped with MSL1 staining in males, but a few bands stain for only SPT5 or MSL1 (Figure 3B control panel and Figure S5A). The presence of SPT5 only bands is not surprising since several genes on the X escape dosage compensation [7]. A possible explanation for the MSL1 only bands will be presented below.

### MSL complex binding persists in the absence of elongation

In order to place SPT5 in the dosage compensation pathway, we focused on two of its most intensively studied roles. First, unphosphorylated SPT5 binds to and pauses RNA polymerase over the transcription start site (TSS). Release from this 5′ pause requires phosphorylation by P-TEFb at multiple sites at the C-termini of both RNA polymerase large subunit and SPT5 [25]–[27]. One way SPT5 might aid dosage compensation is if MSL complex stimulated release of the paused RNAPII/SPT5 complex at male X-linked genes. We refer to this as the Pause Release Model. Extra X-linked transcripts would result from clearing the 5′ end of genes freeing them for additional rounds of initiation. After phosphorylation by P-TEFb, SPT5 switches to a positive elongation factor that accompanies RNA polymerase down the gene. The MSL complex might instead enhance the processive action of SPT5 preventing pausing and/or premature termination as RNAPII moved across X-linked genes (Elongation Model).

The Pause Release model is less appealing because it calls for MSL action at the TSS, when MSL complex is instead predominantly located farther downstream [6]–[8]. One way to explain this discrepancy would be if the MSL complex only fleetingly interacts with P-TEFb or SPT5 at the 5′ end but then travels across the gene with the elongating RNAPII. If true, this model predicts that loss of SPT5 would lower MSL complex occupancy of the male X-chromosome because MSL complex could not enter the body of genes. Unfortunately, we cannot generate *Spt5* null tissue. However, we can approximate that condition by using elongation inhibitors DRB and flavopiridol that block P-TEFb phosphorylation of RNAPII CTD Ser2 and SPT5 that are necessary for pause-release and entry into elongation [23], [28]. After exposure to these drugs, elongating RNAPII continues to the 3′ end of genes, but new RNAPII is trapped at the TSS, effectively stripping gene bodies of RNAPII and SPT5. Treating salivary glands with either inhibitor removed actively elongating RNAPII (Ser2P and Ser5P phosphorylated) from all chromosomes, but paused RNAPII that is Ser5P phosphorylated was unchanged consistent with previous reports [28] (Figure 3A and 3C). The banded SPT5 signal was strongly reduced on all chromosome arms after DRB or flavopiridol treatment (Figure 3B and data not shown). More importantly, the MSL complex staining pattern remained unchanged following inhibitor treatment (Figure 3 and data not shown). This shows that although MSL complex preferentially binds actively transcribed genes, binding persists for some time after the last polymerase has passed. This finding may explain the few loci bound by MSL complex but not SPT5 in untreated animals (Figure S5D and S5E). These may be dosage compensated genes whose developmentally controlled transcription ceased prior to fixation.

Modified histone H3K36me3 is found within active genes with a 3′ bias similar to the MSL complex. This modification may provide one component of MSL targeting specificity through the MSL3 chromodomain [29], but the issue is contentious [4]. We saw no difference in H3K36me3 staining between flavopiridol treated and mock treated tissue (Figure S6) consistent with earlier reports that these inhibitors only modestly lowered H3K36me3 [30].

To examine MSL binding at a higher resolution than is possible with polytenes, we turned to ChIP analysis of male S2 cells. We measured MSL1 binding to the 5′ and 3′ ends of two highly validated target genes after treatment with flavopiridol. If the scarcity of MSL complex at the 5′ ends of X-linked genes was caused by released RNAPII/SPT5 complex quickly carrying it into the body of genes, we might be able to trap MSL complex over the TSS by treatment with P-TEFb kinase inhibitors. The Pause Release Model predicts that flavopiridol treatment should cause the MSL signal to accumulate at the 5′ end of genes with a corresponding loss at the 3′ end. However, just as was seen with the polytene experiments, flavopiridol treatment did not alter MSL complex distribution as measured by ChIP (Figure 3D). These results argue against the Pause Release Model and instead favor the idea that MSL complex acts upon SPT5 during active elongation.

### SPT5 interacts directly with MSL1 PEHE domain

**Figure 4:**
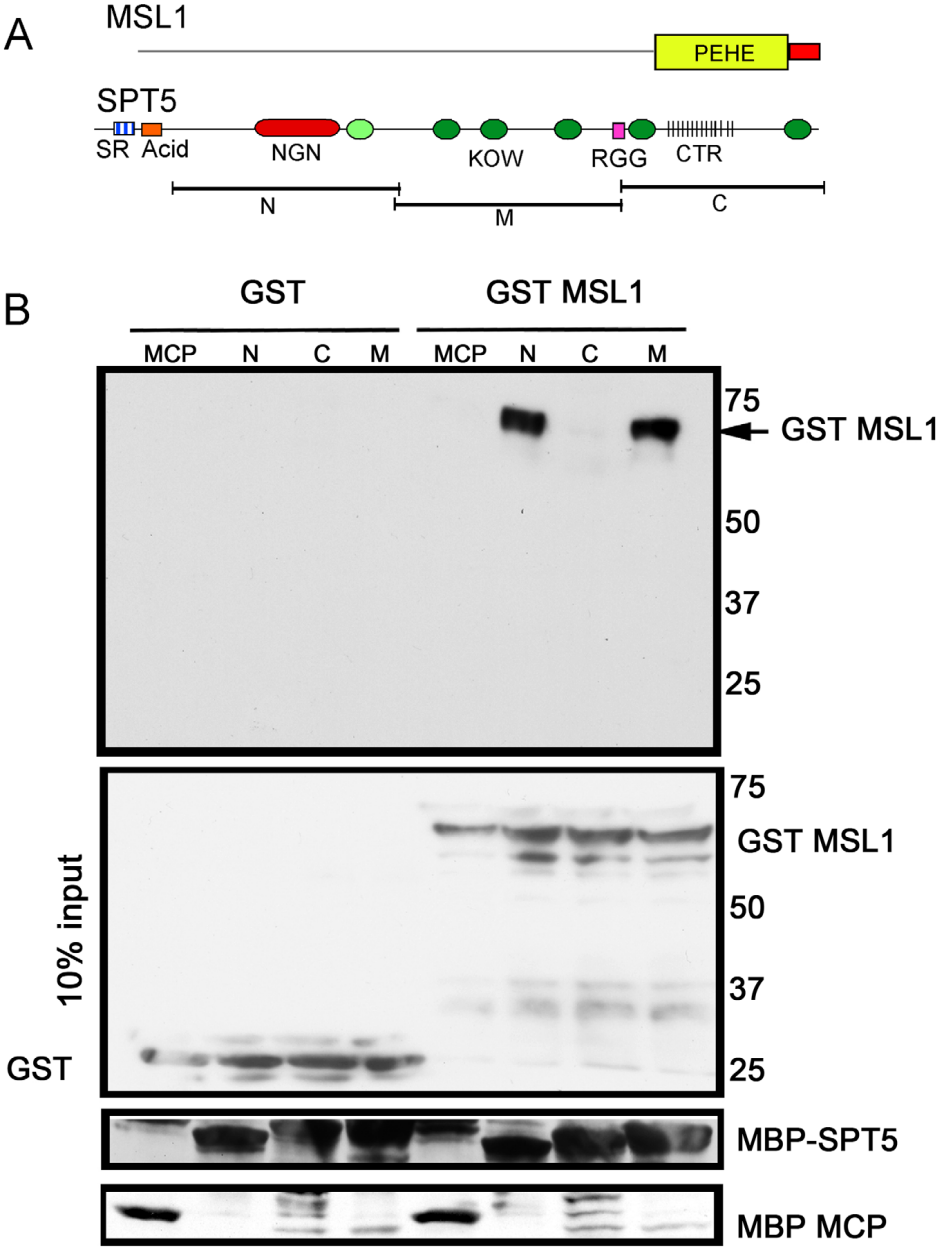
MSL1 PEHE domain physically interacts with SPT5. (A) MSL1 PEHE domain (aa 751–1039) was expressed as GST (Glutathione S-transferase) fusion protein. SPT5 fragments N, M, and C were expressed as MBP (Maltose binding Protein) fusion proteins [16]. (B) Purified SPT5-MBP fragments immobilized on amylose beads were allowed to interact with either GST-MSL1PEHE or GST. After washing, the recovered proteins were analyzed anti-GST Westerns. The membrane was stripped and reprobed to visualize the MBP fusion proteins. The middle input panel corresponds to 10% of the input GST proteins visualized with anti-GST antibodies.

We tested whether the genetic interactions observed between SPT5 and dosage compensation might arise from direct physical contacts. Early attempts to purify intact MSL complex did not recover SPT5 as a partner suggesting that if such interactions occur, they are transient [31], [32]. Dosage compensation in Drosophila is thought to have recruited an ancestral chromatin modifying complex found in most animals by evolving a new targeting strategy to the male X. If true, perhaps any SPT5-MSL interaction predates Drosophila dosage compensation and would be found in the most phylogenetically conserved regions of the complex. We tested the ancient PEHE domain of MSL1 that recruits MSL3 and MOF and forms a functionally critical core of the complex [13]. We asked whether purified subdomains of SPT5 (Figure 4A) could specifically pull down isolated MSL1 PEHE motif. We found specific binding between MSL1 PEHE and the N-terminal (N) and middle fragment (M) SPT5 fragments (Figure 4B). The N fragment contains the NusG-like domain that interacts with the RNAPII clamp domain to encircle the template DNA and makes RNAPII processive [33], [34] and one KOW motif (Figure 4A). The M segment contains additional KOW domains. KOW domains found in other proteins bind either protein or RNA partners [35]. We failed to detect any interactions with the C-terminal region that is phosphorylated at multiple sites by P-TEFb. Although this analysis does not exclude additional contacts between other MSL subunits or *roX* RNAs with regions of SPT5 *in vivo*, the data show that SPT5 and MSL complex have the ability to interact via MSL1 PEHE.

### Other factors found in the screen

Although we focused our analysis on *Spt5*, we wondered if the other modifier mutations found in the screen might identify a new class of factors needed for dosage compensation. We were able to map a few modifiers to previously characterized genes. In the case of deficiencies, we tested whether point mutations of candidate genes could recapitulate the effect of deficiency. That approach showed that *Chromator* was the relevant gene that dominantly suppresses the MSL complex dependent reporter expression in *Df(3L)BSC21*. CHRO is a chromodomain protein that localizes specifically to the interband regions and is implicated in maintaining chromosome structure [36], [37]. Importantly, it copurifies with the MSL complex [31] underscoring the validity of our genetic approach to search for factors involved in dosage compensation. Additionally, we found that a complementation group from the EMS mutagenesis screen fell within the *Df(2R)vg-C* (Figure S1B). This interval contained three strong candidates, *Spt4*, *iswi*, and *Sin3A*. Complementation tests eliminated *iswi*. In eukaryotes, SPT5 usually acts in a complex with SPT4. No point mutations in *Spt4* have been reported in flies, but the gene is not essential in yeast [38]. All the new EMS mutations instead failed to complement a known lesion in *Sin3A* [39], the first indication that the SINA/RPD3 histone deacetylase complex may play a role in dosage compensation. No deficiency removes *Spt5* so this region was not covered in our deficiency screen.

## Discussion

It is possible that dosage compensation in Drosophila is entirely a consequence of the known histone modifications carried out by its subunits, H4K16ac (MOF) and H2BK34ub (MSL2). However, if additional factors are required, new approaches may be needed to identify them. Biochemical purification is challenging due to the very large size of the MSL complex, the presence of the noncoding *roX* RNAs, and the fact that active MSL complex is tightly associated with transcribed chromatin. Extraction methods strong enough to release soluble MSL complex from chromatin may destroy critical contacts with key partners. Genetic approaches also face limitations. If an important partner performs additional functions beyond dosage compensation, mutations would likely be lethal to both sexes masking its interaction with the MSL complex.

We developed an unbiased forward genetic screen able to detect subtle changes in MSL activity that are not large enough to prevent dosage compensation of the male X, but sufficient to alter a sensitive eye pigmentation reporter. This screen implicated *Spt5*, a universally conserved transcription processivity factor for RNAPs, in the MSL pathway [27], [33], [40], [41]. The validity of our approach is illustrated by the identification of mutations in known components involved in the process such as *msl1*, *mle* and *Chro* [13]. The value of a genetic approach to detect protein interactions that may only be stable on actively transcribed chromatin is evident. The Drosophila protein interaction map (DPiM) identified dozens of proteins that are candidate interactors with SPT5 but surprisingly found no stable contacts with subunits of either RNA polymerase II or P-TEFb, the most highly validated partners known from other studies. This search also found no contacts with MSL subunit [42], [43]. The technical difficulty most likely rests with the problem of isolating an enormous complex of many megadaltons tightly tethered to DNA.

Multiple lines of evidence support a role for SPT5 in dosage compensation. The effect of *Spt5* mutations on the *white* eye color reporter was entirely dependent upon the adjacent *roX1* locus that can recruit soluble MSL complex to any location in the genome. *Spt5* mutations had no effect on *white* or *miniwhite* gene expression when not linked to *roX1*. *Spt5* mutations acted on all mosaic *roX1* reporter transgenes regardless of the chromatin environment surrounding the inserts. Interactions of *Spt5* mutants and gain of function *msl1* alleles suggest that SPT5 acts between MSL complex and RNA polymerase. Mutations in *Spt5* selectively reduced male viability under limiting *roX* RNA conditions in a manner comparable to the effect of *mle* mutations. Additionally mutations in *Spt5* partially suppressed the toxic effects of ectopic dosage compensation in females. An independent screen of the Drosophila deficiency collection showed that the *Spt5* phenotype is rare. Removing one allele of almost any transcription related factor had no effect on the eye pigmentation levels of mosaic *roX1* reporters arguing that dosage compensation is particularly sensitive to SPT5 protein levels. Finally, we found that the most ancient and conserved segment of the MSL1 protein physically binds to two different regions of the SPT5 protein consistent with the largely overlapping patterns of chromatin occupancy across the body of X-linked genes.

In eukaryotes SPT5 along with SPT4 forms the DSIF (DRB: 5, 6-dichloro-1-β-D-ribofuranosylbenzimidazole Sensitivity Inducing Factor) [27]. The highly conserved NusG like domain (NGN) docks to RNAP through its interaction with the RNAP clamp domain and closes the cleft where the tightly bent melted DNA template resides preventing RNAP from falling off the template [33] (Figure 1G). The multiple KOW domains may contact either the emerging nascent transcript or other transcription factors. Although some studies indicated that SPT5 acts on a restricted set of genes [44], genome wide ChIP analysis showed that SPT5 and RNAPII colocalize throughout the genome [23], [28]. SPT5 arrests RNAPII near the transcription start site as the short nascent transcript emerges from the enzyme [25]–[27]. The highly regulated release from pause is controlled by the P-TEFb kinase phosphorylating multiple sites near the C-terminus of SPT5 and RNAPII CTD [25], [27], [45].

**Figure 5:**
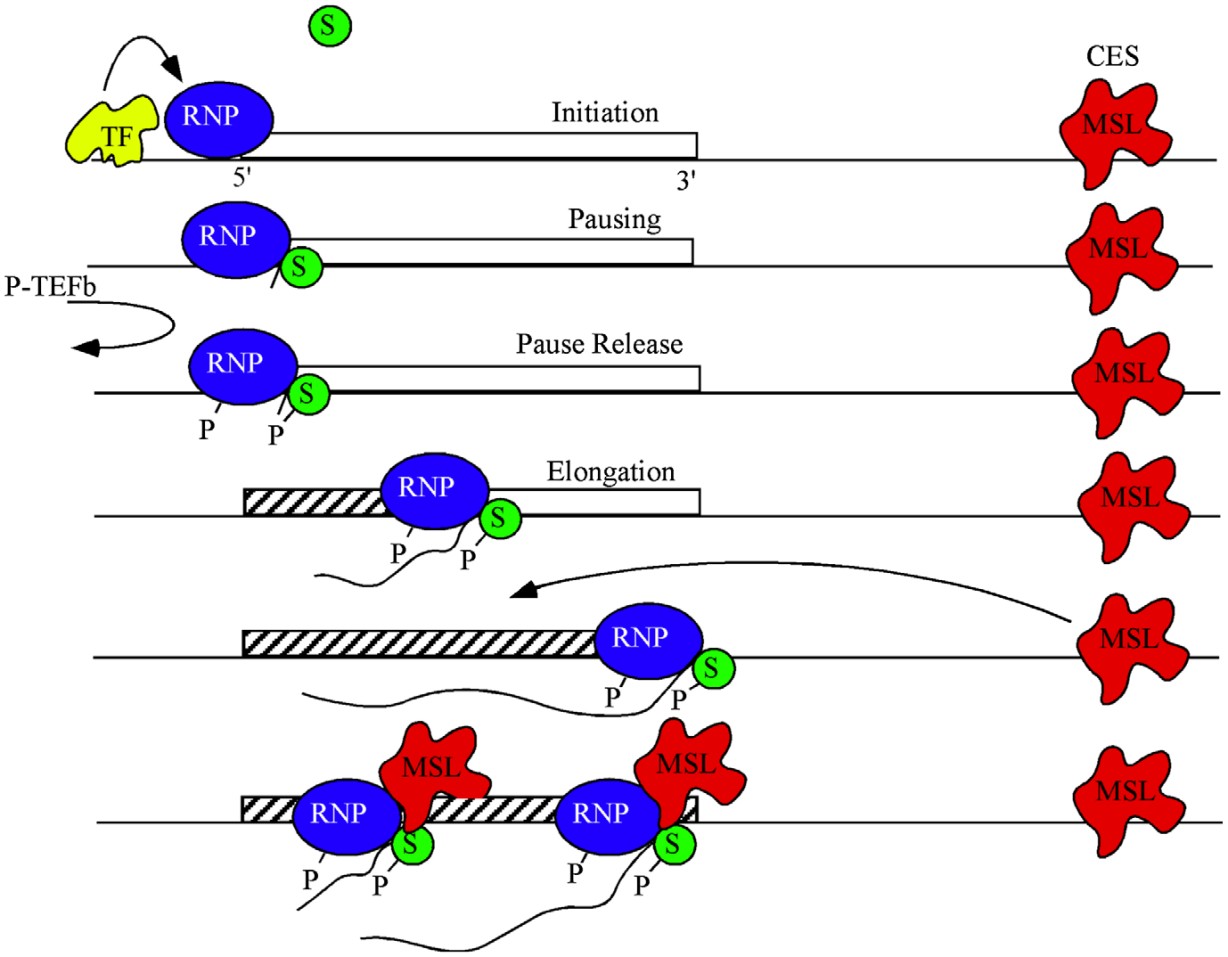
A model for dosage compensation. A highly simplified view separates transcription into phases of initiation controlled by gene-specific transcription factors (yellow), pausing of RNA polymerase II (blue) near the TSS by SPT4/5 (green), and release of pausing when P-TEFb phosphorylates both the CTD of RNAP and SPT5 leading to productive elongation. MSL complex (red) is attracted to the X chromosome by high affinity or chromatin entry sites (CES) scattered along the chromosome. The pioneer RNP may lay down new chromatin marks (hatch) characteristic of active genes. Some feature of active chromatin recruits MSL complex from local CES. During subsequent rounds of transcription MSL complex interacts with SPT5 to promote processivity.

While regulated release from pause was originally described using the highly inducible *hsp70* gene from Drosophila [46], it is now recognized as a widespread step in transcriptional regulation [23], [28], [47]. Although other factors, such as cMyc, stimulate transcription through pause-release of SPT5 [28], our results argue against a similar mechanism operating in Drosophila dosage compensation. MSL complex occupancy is lowest around the TSS and does not depend on continuous association with the elongating RNAPII/SPT5 to be enriched within the gene bodies. Instead we propose that the effect of SPT5 on dosage compensation is downstream of MSL complex recruitment (Figure 5).

MSL complex mediated H4K16ac is enriched within the body of genes and drives decondensation of chromatin possibly facilitating easier passage of RNAPII [5], [48]. It is plausible that within this chromatin domain, SPT5 impacts dosage compensation via its known interactions with SPT6, which eases RNAPII passage by nucleosomal removal [16], [24], [40], [49], [50] and thereby improves the elongation rate of RNAPII. Alternatively, the interaction between MSL complex and SPT5 may increase elongation rates of dosage compensated genes on the X by enhancing RNAPII processivity [33]. We hypothesize that passage of a pioneer RNAPII generates certain transcription-specific epigenetic modifications such as H3K36me3 across a gene. These modifications recruit MSL complex from nearby X-linked sequence specific binding sites called Chromatin Entry Sites (CES) or High Affinity Sites (HAS) [1]. Once recruited, MSL complex is stably bound and stimulates elongation via its transient interaction with the transcribing SPT5/RNAPII (Figure 5). At least one point of this interaction is via the MSL1 PEHE domain and SPT5 NusG like and KOW domains.

While we have focused on the analysis of *Spt5* in this report, our genetic approach also yielded additional candidates. So far, we have mapped two of these to *Chro* and *Sin3A*. CHRO, a chromodomain protein copurifies with the MSL complex [31]. Interestingly, CHRO recruits and localizes with JIL-1, a histone kinase that has also been implicated in dosage compensation [36], [37], [51], [52]. The CHRO/JIL-1 kinase complex is thought to maintain chromosomal integrity [36], [37]. It is conceivable that this complex plays a similar role in maintaining the specialized X-chromatin architecture in male flies. SIN3A, part of the SIN3A/RPD3 histone deacetylase complex is attracted by phosphorylated SPT5 and Ser2 phosphorylated CTD of RNAPII to deacetylate histones in the wake of transcribing RNAPII within the H3K36me3 chromatin domain [53], [54]. Therefore, phosphorylated SPT5, in addition to modulating processivity may also play a role in erasing transcription dependent acetylation via recruiting the SIN3A/RPD3 complex. This serves to suppress spurious transcription initiation from cryptic promoters within the coding region [53], [54]. Alternatively, the SIN3A/RPD3 complex may play a role in MSL complex recruitment to the GAGA element rich MSL recognition elements (MRE) sequences via its interaction with GAGA factor [55]. Further enquiry into the specific role played by these newly identified factors will result in an improved understanding of the mechanism of dosage compensation.

An independent RNAi screen using an MSL complex dependent luciferase expression as a reporter in S2 cells also identified a role for CHRO and SIN3A in dosage compensation [56]. Recovering overlapping cofactors from rather different genetic screens increases confidence that these strategies are identifying authentic components of the dosage compensation pathway. However, SPT5 was not found using the RNAi screen. This is not surprising since a general transcription factor such as SPT5 probably affects the expression of the normalizing control used in luciferase reporter assays. This again highlights the usefulness of an *in vivo* genetic strategy.

Our results provide direct *in vivo* support for the elongation model of dosage compensation by linking the SPT5 elongation factor to the MSL complex [5]. The finding that the *Drosophila* males have ∼1.4 fold more transcriptionally engaged RNAPII at the distal ends of X-linked genes as compared to autosomes also supports the idea of increased elongation [9]. Conversely, a recent report that compared global RNAPII occupancy in males and females found an increase in RNAPII levels across the entire body of the gene including the promoters on male X-linked genes [10]. This observation raises the possibility that dosage compensation may operate at the level of transcription initiation. A caveat of this study is that only a subset of X-linked genes (n = 242) had detectable RNAPII within the body of genes, possibly due to technical difficulties in the ability to detect elongating RNAPII. An alternate explanation for the results is that lowered RNAPII pausing [9], [10] and increased elongation improves RNAPII recycling from the 3′ to 5′ end of genes possibly via gene looping interactions and may be reflected in ChIP seq studies as an increase in RNAPII levels at the promoter. Moreover, Conrad et al postulate that H4K16ac at promoters is the key to dosage compensation. However, H4K16ac at promoters occurs both on male autosomes and all chromosomes in females and is not specific to the male X [48], [57]. On the other hand, H4K16ac within gene bodies is a unique feature of transcribed genes on the male X-chromosome and is therefore an attractive candidate to drive dosage compensation by improving RNAPII passage across the chromatin fibre during elongation.

Mammals also contain a version of the MSL complex composed of MSL1, 2, 3 and MOF, but apparently lacking a large noncoding RNA component and RNA helicase [58], [59]. Like flies, the human MSL complex, is bound within the bodies of genes with a distinct 3′ bias, acetylates histone H4K16 in the body of genes and increases transcription by approximately two fold [58]–[60]. Our results linking the most conserved domain of MSL1 with the conserved transcription elongation factor SPT5 in flies indicate that mammalian MSL complex is likely to also act upon transcription elongation.

## Materials and Methods

### Fly stocks

Mutagenesis was performed as described [13]. Detailed mutagenesis scheme and protocol is included in Text S1. For the deficiency screen the transgenic lines *[w^+^ GMroX1-58D], [w^+^ GMroX1-60F], [w^+^ GMroX1-69C], [w^+^ GMroX1-75C], [w^+^ GMroX1-99F]* and *[w^+^ GMroX1-102C]* were used. The full genotype of Δ*roX1,roX2* stock is *y w roX1^ex6^ Df(1)roX2^52^ [w^+^ cos4Δ4.3]* [19].

### Antibody generation and affinity purification

Plasmids for bacterial expression of MBP fusion SPT5 protein fragments, SPT5-N (aa 112–393), M (aa 389–733) and C (aa 732–1054) were a kind gift from Dr. John Lis [16]. Antibodies were raised by Cocalico Biologicals, Pennsylvania.

### Polytene squashes

Polytene squashes were prepared as described in [61]. Primary antibodies were rabbit anti-MSL1 antibodies (1∶50), guinea pig anti-SPT5 antibodies (1∶100), mouse H5 monoclonal anti-Ser2P RNAP (Covance, 1∶30), mouse H14 monoclonal anti-Ser5P RNAP (Covance, 1∶50) and rabbit anti-H3K36me3 (Invitrogen, 1∶50). Appropriate secondary antibodies were used in combinations that allowed for dual protein localization.

### Chromatin immunoprecipitation

Detailed protocol can be found in Text S1. For inhibitor treatment 500 nM flavopiridol or 100 µM DRB was used. Briefly S2 cells were crosslinked with 1% formaldehyde and nuclei extracted in 15 mM HEPES, 5 mM MgCl2, 0.2 mM EDTA, 0.5 mM EGTA, 10 mM KCl, 350 mM Sucrose, 0.1% Tween 20, 0.5 mM PMSF, 1 mM DTT. Chromatin was sheared to 300–700 bp fragments and pulled down with anti MSL1 antibodies (Gift from M.Kuroda). After several washes eluted chromatin was used. For real time PCR (ABI 7900 qPCR model), SYBR green master mix (ABI), 1 µM primers and 1 µl of input and ChIP DNA was used. Primer sequences are provided in Text S1.

### Protein interaction

MBP-SPT5N, MBP-SPT5M, MBP-SPT5C, the unrelated protein MBP-MCP (MS2 phage coat protein), GST-MSL1 C-terminal domain fusion protein and GST were expressed and isolated from bacteria. Equivalent molar concentrations of MBP proteins bound to amylose beads were incubated with GST proteins. After three washes with 150 mM NaCl, 0.1% NP40 and 20 mM Tris for 10 mins at 4°C, proteins were eluted by boiling in SDS-loading buffer and separated on 8% SDS-polyacrylamide gels. Westerns were performed as described [13]. We used anti-GST antibodies (Sigma) to detect GST and affinity purified guinea pig anti-SPT5 sera to detect MBP-bound SPT5. The proteins were visualized by using appropriate HRP conjugated secondary antibodies (Jackson Immuno) and lunimol reagent (Santa Cruz).

## Supporting Information

Figure S1 A forward genetic screen to identify modifiers of MSL complex activity. (A) Approximately 16,000 males were screened and 48 modifier lines established. (B) Mutations were placed into complementation groups based on recessive lethality. The mutations scored as single hits are recessive lethals, which could be due to the modifier allele or an EMS induced secondary mutation. The mutants scored as viable suppressors lower MSL complex dependent red pigmentation and are homozygous viable. We identified 5 alleles of *spt5*.
(TIF)

Figure S2 Mutations in *Spt5* consistently lower mosaic eye pigmentation independent of autosomal *roX1* insertion site. Shown above are flies hemizygous for the *[GMroX1]/+* transgene at different positions in the genome shown on the side. Flies on the left are wildtype whereas flies on the right are heterozygous for *Spt5^S14F^*. All flies shown are males.
(TIF)

Figure S3 Mutations is *Spt5* do not affect the *white* promoter. Males and female eye pigmentation is shown for the hypomorphic *w* ^a^ and *w* ^e^ alleles with and without *Spt5* mutations. Although *Spt5*/+ heterozygous males show dramatic pigment reductions from the dosage compensation [*w* ^+^ *GMroX1*] mosaic transgenes (Figure 1), the same *Spt5* mutations do not reduce *w* expression when it is not linked to the *roX1* gene. *Spt5* mutations also do not affect the pigmentation of flies carrying unrelated *miniwhite* marked transgenes (data not shown).
(TIF)

Figure S4 Validation of anti SPT5 antibodies. (A) Polytene chromosomes stained with anti-SPT5 antibodies. SPT5 is widely distributed on many sites on the genome. (B) Immunodepletion of anti-SPT5 antibodies with SPT5 fragments- SPT5N, SPT5M and SPT5C results in loss of signal indicating that the antibodies predominantly recognize SPT5. (C) Polytene chromosome spreads prepared from males subjected to 30 min heat shock at 37°C were stained with anti-SPT5 antibodies. In agreement with previous reports [16], [24] upon heat shock, stronger SPT5 bands are observed at the heat shock loci while most other genes lose SPT5. (D) anti-SPT5 antibodies recognize a band approximately 135 kDa on western blots.
(TIF)

Figure S5 SPT5 and MSL1 colocalize extensively on the X-chromosome. Polytene chromosome spreads were stained with antibodies against (A) MSL1 (green) and (B) SPT5 (red). As expected of a general transcription elongation factor SPT5 binds all over the genome. (C) Colocalization of MSL1 and SPT5. (D,E) A closer look at two regions of the X-chromosome. Arrow heads indicates MSL1 only (no SPT5) binding and arrows indicate SPT5 only (no MSL1) binding.
(TIF)

Figure S6 Treatment with Flavopiridol does not affect H3K36me3. (A) Polytene chromosome spreads prepared from salivary glands treated with either DMSO (control panel) or 500 nM Flavopiridol for 30 minutes. Chromosomes were stained with antibodies against H3K36me3. (B) Ser5P RNAPII and SPT5 colocalize on polytenes. After treatment with Flavopiridol, SPT5 is greatly reduced but low levels of SPT5 are found at polytene bands that are positive for Ser5 Phosphorylated RNAPII. (C) A close-up of Ser5P RNAPII and SPT5 colocalization in control and flavopiridol treated samples.
(TIF)

Table S1 Results of Deficiency screen. Six mosaic *roX1* lines were assayed in the deficiency screen (Bloomington Deficiency collection) and the deficiencies that dominantly suppressed the red pigmentation across more than four different mosaic *roX* lines are indicated. Candidate genes that have been mapped within those deficiencies are also shown. Only ten intervals (14/190 deficiencies) assayed had an effect on the eye phenotype indicating that a general suppression of transcription does not lower the expression of the dosage compensation reporter. *Spt5* would be an eleventh locus, but is not uncovered in the Df collection.
(DOC)

Table S2 Lowering *Spt5* decreases male viability. In the absence of both *roX1* and *roX2* males are dead. The male specific lethality can be rescued by an autosomal *GMroX1* transgene. In this assay *roX1-,roX2-; [GMroX1-75C]* virgins were crossed to balanced males carrying the indicated mutations. The male progeny of this cross that are *roX1-,roX2-/Y;mutation/+;[GMroX1-75C]/+* are compared to the nonbalanced female progeny. All the flies therefore carry only one copy of the indicated mutation. The statistical significance was determined by Fisher exact fit test. Figure 2A. Graphical representation of this analysis.
(DOC)

Text S1 Supplementary materials and methods.
(DOC)
