# Supplementary figures and images for: Mutations in the Transcription Elongation Factor SPT5 Disrupt a Reporter for Dosage Compensation in Drosophila
Source: PLoS Genet. 2012 Nov 29;8(11):e1003073. doi: 10.1371/journal.pgen.1003073 (PMC3510053; doi:10.1371/journal.pgen.1003073)

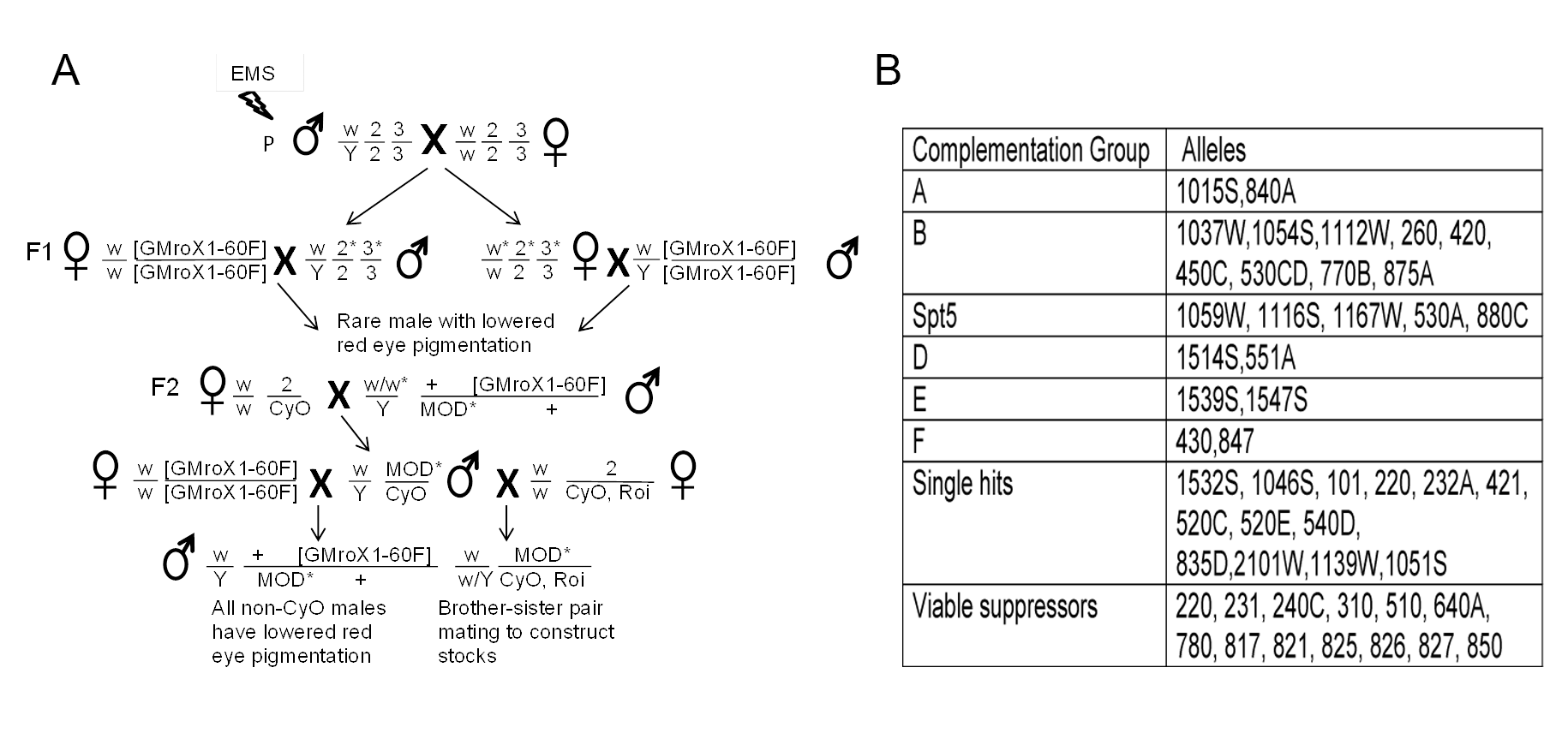

Supplement: Figure S1 — A forward genetic screen to identify modifiers of MSL complex activity. (A) Approximately 16,000 males were screened and 48 modifier lines established. (B) Mutations were placed into complementation groups based on recessive lethality. The mutations scored as single hits are recessive lethals, which could be due to the modifier allele or an EMS induced secondary mutation. The mutants scored as viable suppressors lower MSL complex dependent red pigmentation and are homozygous viable. We identified 5 alleles of spt5. (TIF) [file pgen.1003073.s001.tif]

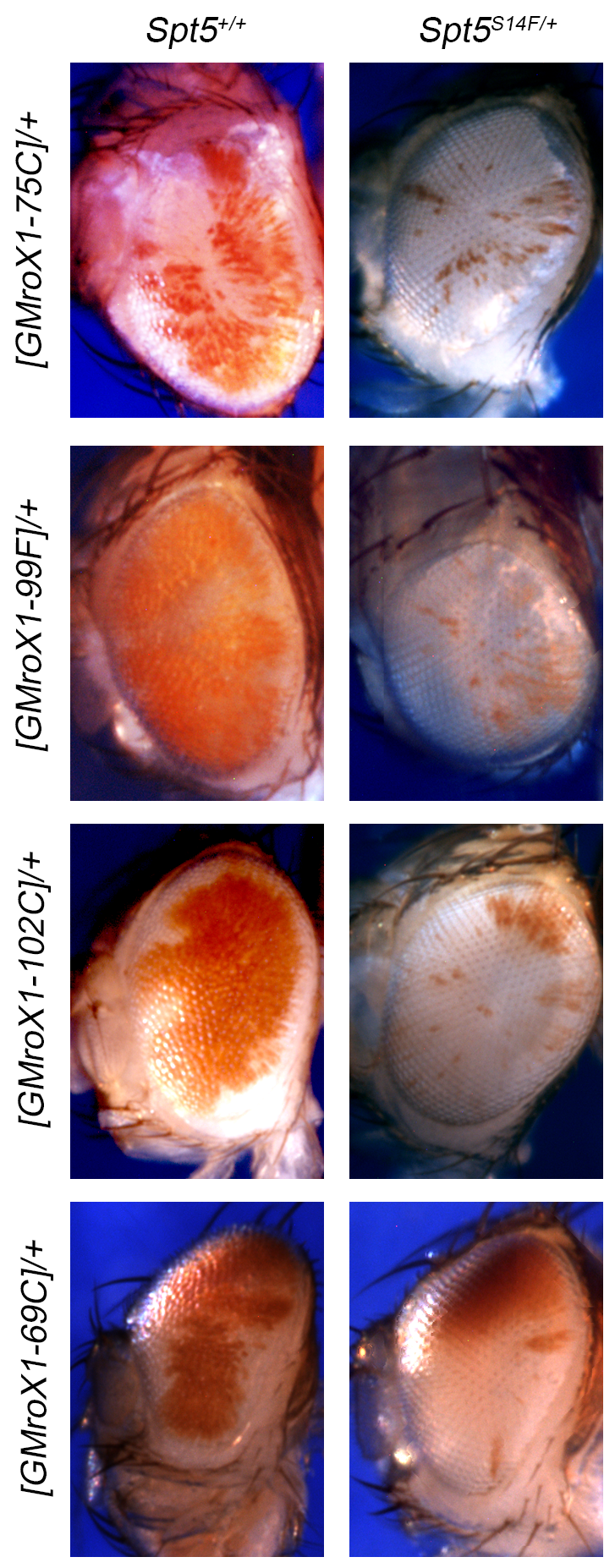

Supplement: Figure S2 — Mutations in Spt5 consistently lower mosaic eye pigmentation independent of autosomal roX1 insertion site. Shown above are flies hemizygous for the [GMroX1]/+ transgene at different positions in the genome shown on the side. Flies on the left are wildtype whereas flies on the right are heterozygous for Spt5S14F. All flies shown are males. (TIF) [file pgen.1003073.s002.tif]

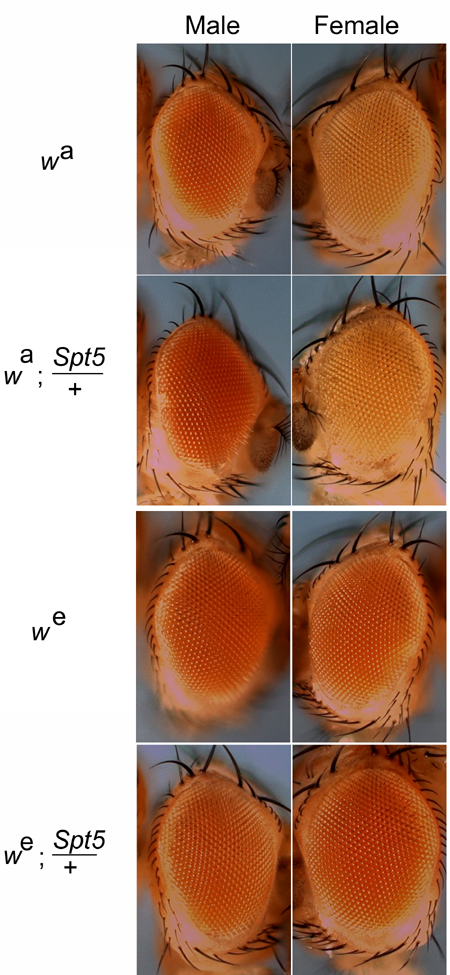

Supplement: Figure S3 — Mutations is Spt5 do not affect the white promoter. Males and female eye pigmentation is shown for the hypomorphic w a and w e alleles with and without Spt5 mutations. Although Spt5/+ heterozygous males show dramatic pigment reductions from the dosage compensation [w + GMroX1] mosaic transgenes (Figure 1), the same Spt5 mutations do not reduce w expression when it is not linked to the roX1 gene. Spt5 mutations also do not affect the pigmentation of flies carrying unrelated miniwhite marked transgenes (data not shown). (TIF) [file pgen.1003073.s003.tif]

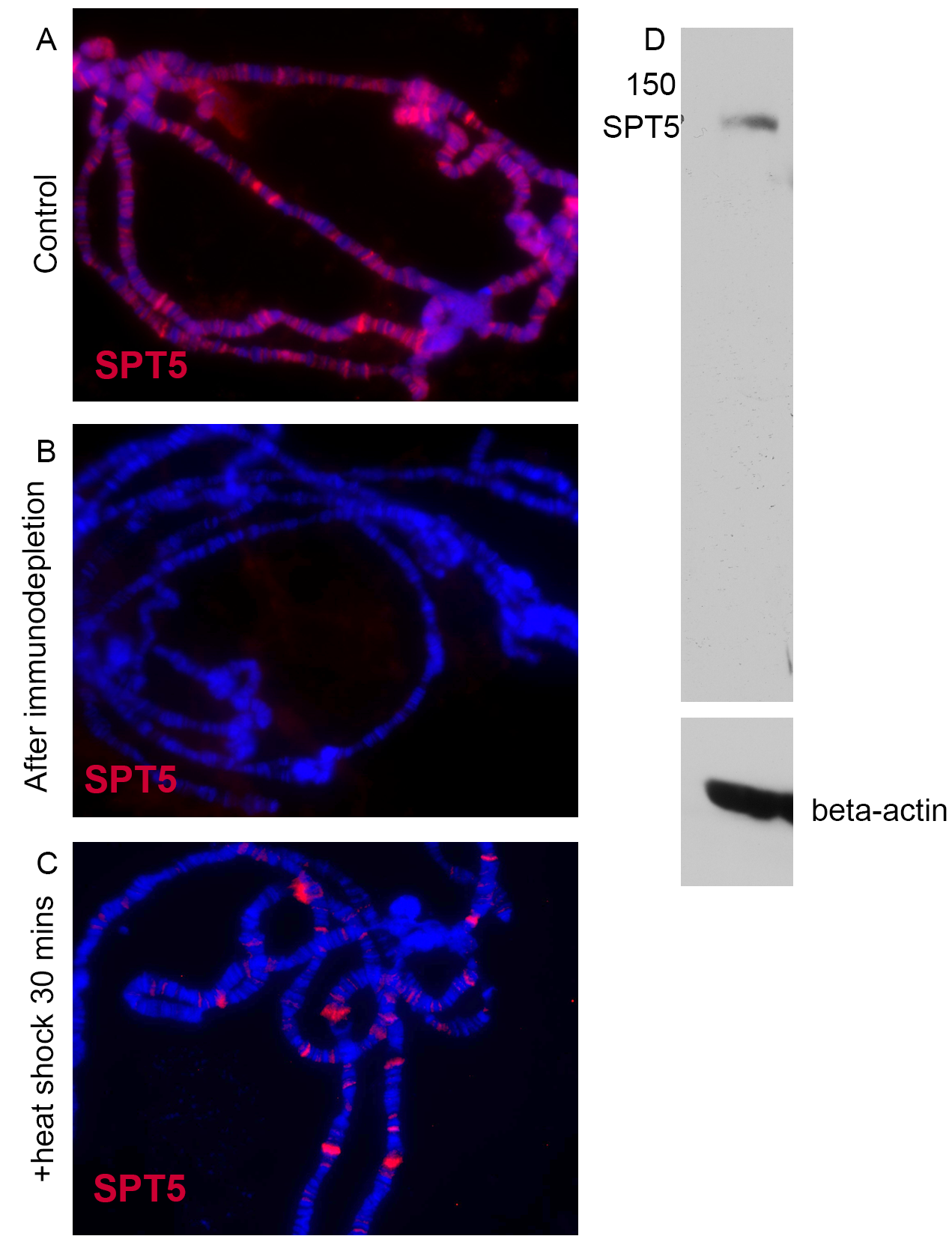

Supplement: Figure S4 — Validation of anti SPT5 antibodies. (A) Polytene chromosomes stained with anti-SPT5 antibodies. SPT5 is widely distributed on many sites on the genome. (B) Immunodepletion of anti-SPT5 antibodies with SPT5 fragments- SPT5N, SPT5M and SPT5C results in loss of signal indicating that the antibodies predominantly recognize SPT5. (C) Polytene chromosome spreads prepared from males subjected to 30 min heat shock at 37°C were stained with anti-SPT5 antibodies. In agreement with previous reports [16], [24] upon heat shock, stronger SPT5 bands are observed at the heat shock loci while most other genes lose SPT5. (D) anti-SPT5 antibodies recognize a band approximately 135 kDa on western blots. (TIF) [file pgen.1003073.s004.tif]

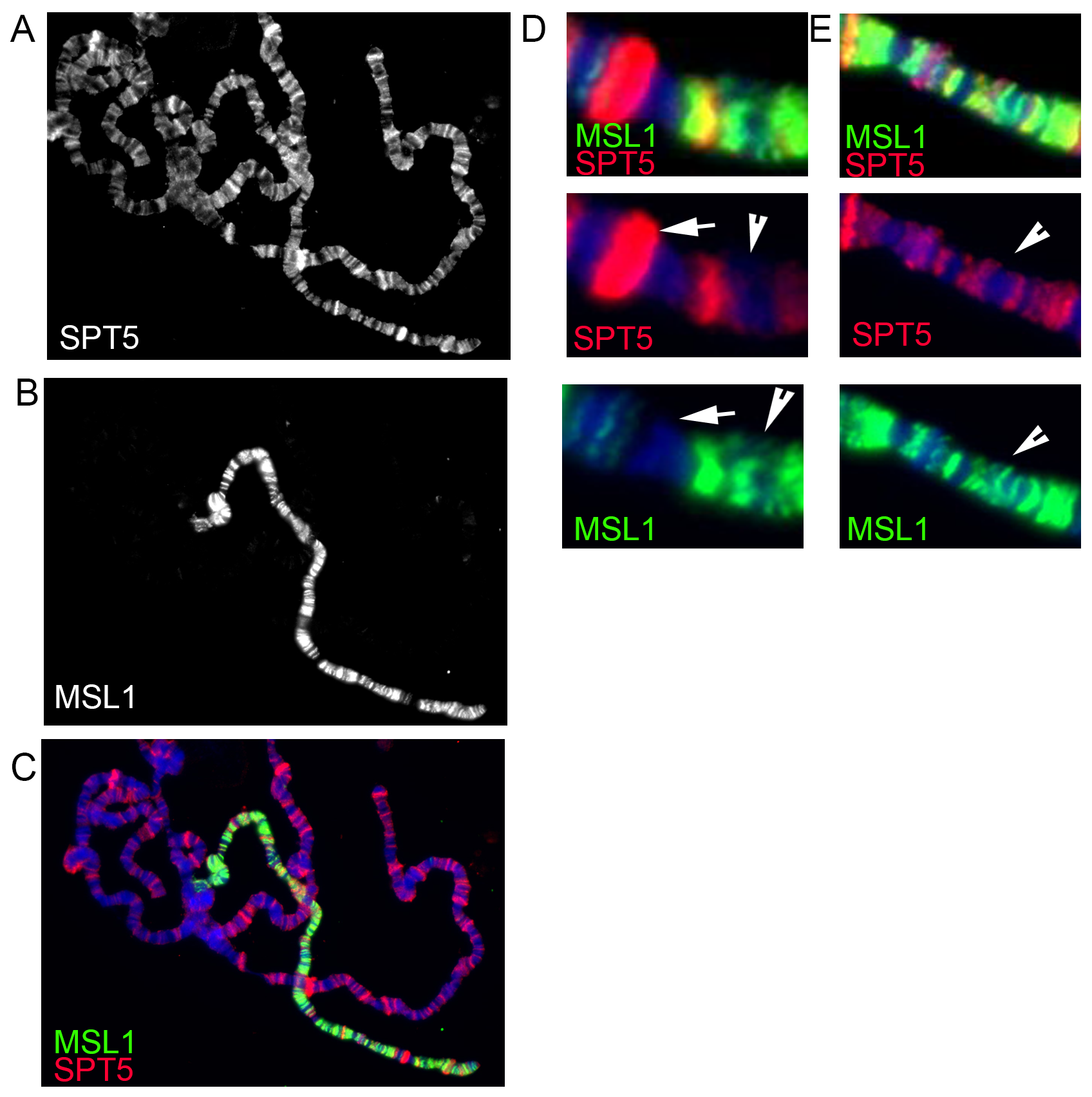

Supplement: Figure S5 — SPT5 and MSL1 colocalize extensively on the X-chromosome. Polytene chromosome spreads were stained with antibodies against (A) MSL1 (green) and (B) SPT5 (red). As expected of a general transcription elongation factor SPT5 binds all over the genome. (C) Colocalization of MSL1 and SPT5. (D,E) A closer look at two regions of the X-chromosome. Arrow heads indicates MSL1 only (no SPT5) binding and arrows indicate SPT5 only (no MSL1) binding. (TIF) [file pgen.1003073.s005.tif]

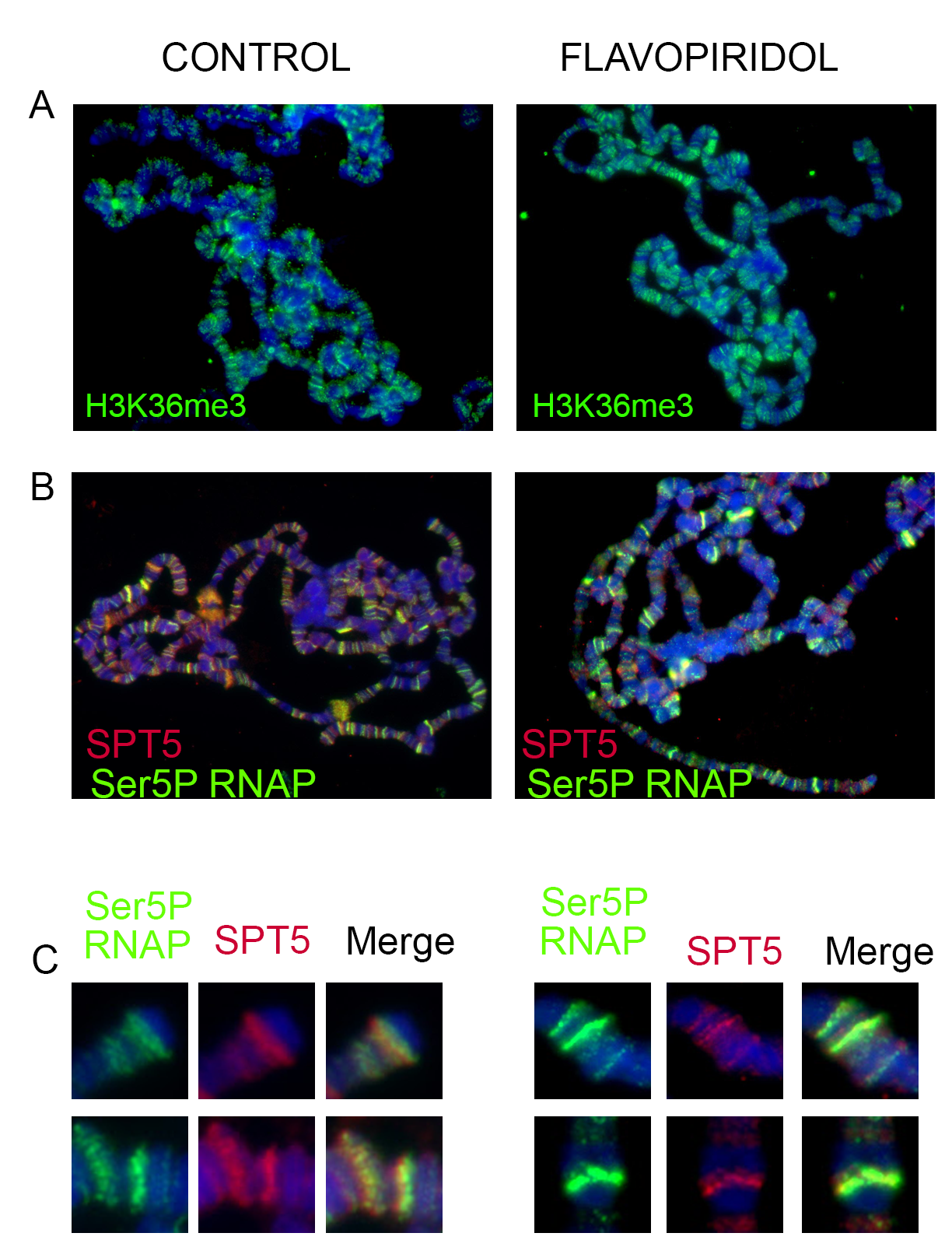

Supplement: Figure S6 — Treatment with Flavopiridol does not affect H3K36me3. (A) Polytene chromosome spreads prepared from salivary glands treated with either DMSO (control panel) or 500 nM Flavopiridol for 30 minutes. Chromosomes were stained with antibodies against H3K36me3. (B) Ser5P RNAPII and SPT5 colocalize on polytenes. After treatment with Flavopiridol, SPT5 is greatly reduced but low levels of SPT5 are found at polytene bands that are positive for Ser5 Phosphorylated RNAPII. (C) A close-up of Ser5P RNAPII and SPT5 colocalization in control and flavopiridol treated samples. (TIF) [file pgen.1003073.s006.tif]
