# Supplementary material for: Mutations in the Transcription Elongation Factor SPT5 Disrupt a Reporter for Dosage Compensation in Drosophila
Source: PLoS Genet. 2012 Nov 29;8(11):e1003073. doi: 10.1371/journal.pgen.1003073 (PMC3510053; doi:10.1371/journal.pgen.1003073)
Supplement: Table S1 — Results of Deficiency screen. Six mosaic roX1 lines were assayed in the deficiency screen (Bloomington Deficiency collection) and the deficiencies that dominantly suppressed the red pigmentation across more than four different mosaic roX lines are indicated. Candidate genes that have been mapped within those deficiencies are also shown. Only ten intervals (14/190 deficiencies) assayed had an effect on the eye phenotype indicating that a general suppression of transcription does not lower the expression of the dosage compensation reporter. Spt5 would be an eleventh locus, but is not uncovered in the Df collection. (DOC) [file pgen.1003073.s007.doc]

| Cytology | Deficiency | Effect | Candidate |
| --- | --- | --- | --- |
| 21A1;21B7--8 | Df(2L)net-PMF |  |  |
| 21B7;21C2 | Df(2L) [BSC106](http://flybase.org/reports/FBab0038756.html) |  |  |
| 21C3--4;21C6--8 | Df(2L)BSC16 | Whiter eyes |  |
| 21D1--2;22B2--3 | Df(2L)ast2 |  |  |
| 22A2--3;22D5--E1 | Df(2L)dp-79b |  |  |
| 22D4;22E1 | Df(2L) [Exel7010](http://flybase.org/reports/FBab0037896.html) |  |  |
| 22E4--F2;22F3--23A1 | Df(2L) [dpp[d14]](http://flybase.org/reports/FBab0001786.html) |  |  |
| 22F4;23C3 | [Df(2L)C144](http://flybase.org/reports/FBab0022184.html) |  |  |
| 23C1--2;23E1--2 | Df(2L)JS17 |  |  |
| 23E5;23F3 | Df(2L) [Exel7016](http://flybase.org/reports/FBab0037900.html) |  |  |
| 24A2;24D4 | Df(2L)ed1 |  |  |
| [24C3--25A2](http://flybase.org/cgi-bin/txtbrowse_fb.html?xfieldname1=CLOC&group=yes&objtype=gene cytogene tRNA ncRNA snRNA snoRNA miRNA rRNA transposable_element_insertion_site cytoins deleted_segment cytodeleted_segment duplicated_segment cytoduplicated_segment&xfield1=24C3--25A2) | Df(2L)ed-dp |  |  |
| 25C1;25C4 | Df(2L)BSC110 |  |  |
| 25C4;25C8 | Df(2L)BSC109 |  |  |
| 25D2--4;26B2--5 | Df(2L) [cl-h3](http://flybase.org/reports/FBab0001758.html) |  |  |
| 25F3--26A1;26D3--11 | Df(2L) [E110](http://flybase.org/reports/FBab0001456.html) |  |  |
| 26D3--E1;26F4--7 | Df(2L)BSC6 |  |  |
| 26F6;27B1 | Df(2L) E[xel7027](http://flybase.org/reports/FBab0037907.html) |  |  |
| [27A--28A](http://flybase.org/cgi-bin/txtbrowse_fb.html?xfieldname1=CLOC&group=yes&objtype=gene cytogene tRNA ncRNA snRNA snoRNA miRNA rRNA transposable_element_insertion_site cytoins deleted_segment cytodeleted_segment duplicated_segment cytoduplicated_segment&xfield1=27A--28A) | Df(2L) Dwee-δ5 | Whiter eyes |  |
| 27C1--2;28A | Df(2L) [spd[j2]](http://flybase.org/reports/FBab0024845.html) |  |  |
| 27C2--3;27C4--5 | Df(2L) [Dwee1-W05](http://flybase.org/reports/FBab0026801.html) |  |  |
| 28A5--B1;28C1--9 | Df(2L)XE-2750 |  |  |
| 28C3;28D3 | Df(2L)BSC142 |  |  |
| 28D2--28E1;28E5 | Df(2L) [Trf-C6R31](http://flybase.org/reports/FBab0022203.html) |  |  |
| 28E4--7;29B2--C1 | Df(2L) [TE29Aa-11](http://flybase.org/reports/FBab0001569.html) |  |  |
| 28F5;29B1 | Df(2L)BSC111 |  |  |
| 29C1--2;30C8--9 | Df(2L) [N22-14](http://flybase.org/reports/FBab0022196.html) |  |  |
| 30C3--5;30F1 | Df(2L)BSC17 |  |  |
| 30D--30F;31F | Df(2L) [Mdh](http://flybase.org/reports/FBab0001497.html) |  |  |
| 31B;32A | Df(2L)J2 |  |  |
| 31B1;31D9 | Df(2L) BSC143 |  |  |
| 31F5;32B3 | Df(2L) [Exel8026](http://flybase.org/reports/FBab0037960.html) |  |  |
| 32B1;32C1 | Df(2L) [Exel7049](http://flybase.org/reports/FBab0037920.html) |  |  |
| 32C1;32C1 | Df(2L) BSC145 |  |  |
| 32D1;32D4--E1 | Df(2L)BSC36 |  |  |
| 32D1;32F1--3 | Df(2L) [FCK-20](http://flybase.org/reports/FBab0028902.html) |  |  |
| 32F1--3;33F1--2 | Df(2L)Prl |  |  |
| 34A3;34B7--9 | Df(2L)BSC30 |  |  |
| 34B12--C1;35B10--C1 | Df(2L)b87e25 |  |  |
| 34C1;34C6 | Df(2L)BSC147 |  |  |
| 35B4--6;35F1--7 | Df(2L) [TE35BC-24](http://flybase.org/reports/FBab0001606.html) |  |  |
| 35D1;36A6--7 | Df(2L)r10 |  |  |
| 35F--36A;36D | Df(2L) [cact-255rv64](http://flybase.org/reports/FBab0022223.html) |  |  |
| 36C2--4;37B9--C1 | Df(2L) [TW137](http://flybase.org/reports/FBab0001645.html) |  |  |
| 37B2--12;38D2--5 | Df(2L)pr-A16 |  |  |
| 38A6-B1;40A4-B1 | Df(2L)TW161 |  |  |
| 40A5;40E5 | Df(2L)BSC151 |  |  |
| 41A;41A | Df(2R)M41A |  |  |
| 42A1--2;42E6--F1 | [Df(2R)nap9](http://flybase.org/reports/FBab0002190.html) |  |  |
| 42B3--5;43E15--18 | [Df(2R)ST1](http://flybase.org/reports/FBab0002037.html) |  |  |
| 42C2--7;43D1--7 | [Df(2R)Drl[rv17]](http://flybase.org/reports/FBab0001440.html) |  |  |
| 42D1;43E6 | Df(2R)Drl[rv7] |  |  |
| 42E;44C | [Df(2R)cn9](http://flybase.org/reports/FBab0002170.html) |  |  |
| 43C1--7;43E15--18 | [Df(2R)tor-rx6](http://flybase.org/reports/FBab0002230.html) |  |  |
| 43F;44D3--8 | [Df(2R)H3C1](http://flybase.org/reports/FBab0024375.html) |  |  |
| 44D1-2;44F12-45A1 | [Df(2R)H3E1](http://flybase.org/reports/FBab0024377.html) |  |  |
| 44F12;45D6-E3 | [Df(2R)Np5](http://flybase.org/reports/FBab0024011.html) |  |  |
| 45A6--7;45E2--3 | [Df(2R)w45-30n](http://flybase.org/reports/FBab0002275.html) |  |  |
| 45D3--4;45F2--6 | [Df(2R)BSC29](http://flybase.org/reports/FBab0029979.html) |  |  |
| 45F6;46B4 | [Df(2R)BSC132](http://flybase.org/reports/FBab0044814.html) |  |  |
| 46A;46C | [Df(2R)B5](http://flybase.org/reports/FBab0010225.html) |  |  |
| 46C;47A1 | [Df(2R)X1](http://flybase.org/reports/FBab0002048.html) |  |  |
| 46D7-9;47F15-16 | Df(2R)stan1 |  |  |
| 47D5-7;48A3-B2 | [Df(2R)en-A](http://flybase.org/reports/FBab0002173.html) |  |  |
| 48A3--4;48C6--8 | [Df(2R)en30](http://flybase.org/reports/FBab0002176.html) |  |  |
| 48E;49A | [Df(2R)CB21](http://flybase.org/reports/FBab0024848.html) |  |  |
| 49A4-13;49E7-F1 | Df(2R)vg-C | Whiter eyes | *Sin3A* |
| 49B5;49B12 | Df(2R)[Exel7121](http://flybase.org/reports/FBab0038032.html) | Whiter eyes | *Sin3A* |
| 49C1;50D3-5 | [Df(2R)CX1](http://flybase.org/reports/FBab0022242.html) |  |  |
| 50D1;50D2--7 | [Df(2R)BSC18](http://flybase.org/reports/FBab0029741.html) |  |  |
| 50D4;50E4 | [Df(2R)Exel7130](http://flybase.org/reports/FBab0038037.html) |  |  |
| 50E1;50E6 | [Df(2R)BSC134](http://flybase.org/reports/FBab0044840.html) |  |  |
| 50E4;50F6 | [Df(2R)Exel7131](http://flybase.org/reports/FBab0038038.html) |  |  |
| 50E6--F1;51E2--4 | [Df(2R)BSC11](http://flybase.org/reports/FBab0029712.html) |  |  |
| 51D3--8;52F5--9 | [Df(2R)Jp1](http://flybase.org/reports/FBab0001967.html) |  |  |
| 54C10;54D5 | [Df(2R)Exel7149](http://flybase.org/reports/FBab0038048.html) |  |  |
| 54D1--2;54E5--7 | [Df(2R)14H10Y-53](http://flybase.org/reports/FBab0029942.html) |  |  |
| 54E8--F1;55B9--C1 | [Df(2R)Pcl7B](http://flybase.org/reports/FBab0002023.html) |  |  |
| 55A;55F | [Df(2R)PC4](http://flybase.org/reports/FBab0002014.html) | Whiter eyes |  |
| 55B8;55E3 | [Df(2R)ED3636](http://flybase.org/reports/FBab0034911.html) |  |  |
| 55E2;55E10 | [Df(2R)Exel7157](http://flybase.org/reports/FBab0038052.html) |  |  |
| 55E2--4;56C1--11 | [Df(2R)P34](http://flybase.org/reports/FBab0002011.html) | Whiter eyes |  |
| 56F11;56F16 | [Df(2R)Exel7162](http://flybase.org/reports/FBab0038054.html) |  |  |
| 57B19-C1;57E1-6 | [Df(2R)AA21](http://flybase.org/reports/FBab0004927.html) |  |  |
| 57D2--8;58D1 | [Df(2R)Egfr5](http://flybase.org/reports/FBab0001960.html) |  |  |
| 58D1-2:59A | [Df(2R)X58-12](http://flybase.org/reports/FBab0022257.html) |  |  |
| [59A1--59D4](http://flybase.org/cgi-bin/txtbrowse_fb.html?xfieldname1=CLOC&group=yes&objtype=gene cytogene tRNA ncRNA snRNA snoRNA miRNA rRNA transposable_element_insertion_site cytoins deleted_segment cytodeleted_segment duplicated_segment cytoduplicated_segment&xfield1=59A1--59D4) | Df(2R)59AD |  |  |
| 59B;59D8--E1 | [Df(2R)vir130](http://flybase.org/reports/FBab0024859.html) |  |  |
| [59D5--60B8](http://flybase.org/cgi-bin/txtbrowse_fb.html?xfieldname1=CLOC&group=yes&objtype=gene cytogene tRNA ncRNA snRNA snoRNA miRNA rRNA transposable_element_insertion_site cytoins deleted_segment cytodeleted_segment duplicated_segment cytoduplicated_segment&xfield1=59D5--60B8) | Df(2R)or-BR6 |  |  |
| 60C4--60C6;60D9 | [Df(2R)Px2](http://flybase.org/reports/FBab0002027.html) |  |  |
| [60E6--60F2](http://flybase.org/cgi-bin/txtbrowse_fb.html?xfieldname1=CLOC&group=yes&objtype=gene cytogene tRNA ncRNA snRNA snoRNA miRNA rRNA transposable_element_insertion_site cytoins deleted_segment cytodeleted_segment duplicated_segment cytoduplicated_segment&xfield1=60E6--60F2) | Df(2R)ES1 |  |  |
| 60F1;60F5 | [Df(2R)Kr10](http://flybase.org/reports/FBab0001981.html) |  |  |
| 61A;61D3 | Df(3L) [emc-E12](http://flybase.org/reports/FBab0002367.html) |  |  |
| 61C5--8;62A8 | Df(3L)[Ar14-8](http://flybase.org/reports/FBab0010230.html) |  |  |
| 62A10--B1;62D2--5 | Df(3L) [Aprt-1](http://flybase.org/reports/FBab0023593.html) |  |  |
| 62B7;62E5-6 | Df(3L)R-G7 |  |  |
| 62F;63D | Df(3L)M21 |  |  |
| 63C2;63F7 | Df(3L)HR119 |  |  |
| 63E6--9;64A8--9 | Df(3L)[GN34](http://flybase.org/reports/FBab0002319.html) |  |  |
| 63F6-7;64C8-9 | Df(3L)GN24 |  |  |
| 64C;65C | Df(3L) [ZN47](http://flybase.org/reports/FBab0000006.html) | Whiter eyes |  |
| 64E;65C1--3 | Df(3L)XAS96 |  |  |
| 65A7--11;65C1--3 | Df(3L)CH12 |  |  |
| 64D1--2;65C3 | Df(3L)CH20 | Whiter eyes |  |
| [65A2--65E1](http://flybase.org/cgi-bin/txtbrowse_fb.html?xfieldname1=CLOC&group=yes&objtype=gene cytogene tRNA ncRNA snRNA snoRNA miRNA rRNA transposable_element_insertion_site cytoins deleted_segment cytodeleted_segment duplicated_segment cytoduplicated_segment&xfield1=65A2--65E1) | Df(3L)XD198,e(1) |  |  |
| 65D4--5;65E4--6 | Df(3L) [BSC27](http://flybase.org/reports/FBab0029948.html) |  |  |
| 65E1--12;66B1--2 | Df(3L) [RM5-2](http://flybase.org/reports/FBab0026857.html) |  |  |
| 66A17--20;66C1--5 | Df(3L)ZP1 |  |  |
| [66B12--66D4](http://flybase.org/cgi-bin/txtbrowse_fb.html?xfieldname1=CLOC&group=yes&objtype=gene cytogene tRNA ncRNA snRNA snoRNA miRNA rRNA transposable_element_insertion_site cytoins deleted_segment cytodeleted_segment duplicated_segment cytoduplicated_segment&xfield1=66B12--66D4) | Df(3L)BSC13, rho^ve-1^ e^1^ |  |  |
| 66B8--9;66C9--10 | Df(3L) [66C-G28](http://flybase.org/reports/FBab0022326.html) |  |  |
| 66D10--11;66E1--2 | Df(3L) [h-i22](http://flybase.org/reports/FBab0002374.html) |  |  |
| 66E1--6;66F1--6 | Df(3L)[Scf-R6](http://flybase.org/reports/FBab0027420.html) |  |  |
| 66F5;67B1 | Df(3L) [29A6](http://flybase.org/reports/FBab0002286.html) |  |  |
| 67A2;67D11--13 | Df(3L) [AC1](http://flybase.org/reports/FBab0002292.html) |  |  |
| [67E5--68B4](http://flybase.org/cgi-bin/txtbrowse_fb.html?xfieldname1=CLOC&group=yes&objtype=gene cytogene tRNA ncRNA snRNA snoRNA miRNA rRNA transposable_element_insertion_site cytoins deleted_segment cytodeleted_segment duplicated_segment cytoduplicated_segment&xfield1=67E5--68B4) | [Df(3L)lxd6](http://flybase.org/reports/FBab0002387.html) |  |  |
| 68A2--3;69A1--3 | Df(3L) [vin5](http://flybase.org/reports/FBab0002457.html) |  |  |
| 68C8--11;69B4--5 | Df(3L)vin7 |  |  |
| 69A2;69D1 | Df(3L)F10 |  |  |
| 69A4--5;69D4--6 | Df(3L)eyg[C1] |  |  |
| 69D4--5;69F5--7 | Df(3L)[BSC10](http://flybase.org/reports/FBab0029692.html) |  |  |
| 70A1--2;70C3--4 | In(3LR)C190^L^Ubx^42TR^ |  |  |
| 70C2--6;70E1 | Df(3L)[fz-CAL5](http://flybase.org/reports/FBab0002369.html) |  |  |
| 70D2--3;71E4--5 | Df(3L) [fz-M21](http://flybase.org/reports/FBab0002373.html) |  |  |
| 71F1--4;72D1--10 | Df(3L)brm11 | Whiter eyes |  |
| 72D2;72D5 | Df(3L)th117 |  |  |
| 72C1--D1;73A3--4 | Df(3L)[st-f13](http://flybase.org/reports/FBab0002412.html) |  |  |
| 73A3;74F | Df(3L)[81k19](http://flybase.org/reports/FBab0000011.html) |  |  |
| 74D3--75A1;75B2--5 | Df(3L)[BSC8](http://flybase.org/reports/FBab0029684.html) |  |  |
| 75A6--7;75C1--2 | Df(3L)[W10](http://flybase.org/reports/FBab0002357.html) |  |  |
| 75B4-7;75E2 | Df(3L)Cat |  |  |
| 75F10--11;76A1—5 | Df(3L)[fz2](http://flybase.org/reports/FBab0028848.html) |  |  |
| 76A7--B1;76B4--5 | Df(3L)[BSC20](http://flybase.org/reports/FBab0029835.html) |  |  |
| 76B4;77B | Df(3L)XS533 |  |  |
| 77A1;77D1 | Df(3L) [rdgC-co2](http://flybase.org/reports/FBab0002392.html) |  |  |
| 77B--C;77F--78A | Df(3L)ri-79c |  |  |
| [77F3--78C9](http://flybase.org/cgi-bin/txtbrowse_fb.html?xfieldname1=CLOC&group=yes&objtype=gene cytogene tRNA ncRNA snRNA snoRNA miRNA rRNA transposable_element_insertion_site cytoins deleted_segment cytodeleted_segment duplicated_segment cytoduplicated_segment&xfield1=77F3--78C9) | Df(3L)ME107,mwh[1]red[1]e[1] |  |  |
| 78C5--6;78E3--79A1 | Df(3L) Pc-2q |  |  |
| 79D3--E1;79F3--6 | Df(3L)HD1 |  |  |
| 79E5--F1;80A2--3 | Df(3L)[BSC21](http://flybase.org/reports/FBab0029836.html) | Whiter eyes | *Chromator* |
| 79F-80A | [Df(3L)Δ1AK](http://flybase.org/reports/FBst0004370.html) | Whiter eyes | *Chromator* |
| [81F3--82F7](http://flybase.org/cgi-bin/txtbrowse_fb.html?xfieldname1=CLOC&group=yes&objtype=gene cytogene tRNA ncRNA snRNA snoRNA miRNA rRNA transposable_element_insertion_site cytoins deleted_segment cytodeleted_segment duplicated_segment cytoduplicated_segment&xfield1=81F3--82F7) | Df(3R)ME15 |  |  |
| 81F4--5;83A | Df(3R)2-2 |  |  |
| 81Fa;81Fb | Df(3R)4-75 |  |  |
| 82F8--10;83A1--3 | Df(3R)e1025-14 |  |  |
| 83C1--2;84B1--2 | Df(3R)Tpl10 |  |  |
| 83E1--2;84A5 | Df(3R)WIN11 |  |  |
| 83E1--2;84A5 | Df(3R)e-N19 |  |  |
| 84A6--B1;84D4--D9 | Df(3R)roe |  |  |
| 84C8--D1;84F6--7 | Df(3R)[dsx29](http://flybase.org/reports/FBab0002759.html) |  |  |
| 84D4--6;85B6 | Df(3R)p712 | Whiter eyes |  |
| 85A1-2;85B8-C1 | Df(3R) p-XT103 |  |  |
| 85B7;85D15 | Df(3R) [BSC24](http://flybase.org/reports/FBab0029839.html) |  |  |
| 85D11-14;85F6 | Df(3R)by62 |  |  |
| 85D8--12;85E7--F1 | Df(3R)by10 |  |  |
| 86C1;87B1--5 | Df(3R)M-Kx1 |  |  |
| 86D9;87C3-4 | Df(3R)86D9;87C3-4 |  |  |
| 87B4;87D7--8 | Df(3R)kar-H10 |  |  |
| 87C7--8;87E5--6 | Df(3R)kar-Sz11 |  |  |
| 87E1--2;87F11--12 | Df(3R) [l26c](http://flybase.org/reports/FBab0002835.html) |  |  |
| 87F12--14;88C1--3 | Df(3R)red[3l](http://flybase.org/reports/FBab0002879.html) |  |  |
| 88B1;88D3--4 | Df(3R)red1 |  |  |
| 88E7--13;89A1 | Df(3R)ea |  |  |
| 88F7--89A2;89A11--13 | Df(3R)Po4 |  |  |
| 89A12;89B6 | Df(3R)Exel7328 |  |  |
| 89A8;89B1 | Df(3R)Exel7327 |  |  |
| 89B7--8;89E7 | Df(3R)P115 |  |  |
| 89E1--F4;91B1--B2 | Df(3R)DG2 |  |  |
| 89E3--4;90A1--7 | Df(3R)C4 |  |  |
| 90F1--F4;91F5 | Df(3R)Cha7 |  |  |
| 91F1--2;92D3--6 | Df(3R)Dl-BX12 | Whiter eyes |  |
| 92B3;92F13 | Df(3R)H-B79 |  |  |
| 93B6--7;93D2 | Df(3R)e-R1 |  |  |
| 93C6;94A1--4 | Df(3R)e-GC3 |  |  |
| 93E--F;94C--D | Df(3R)[5C1](http://flybase.org/reports/FBab0026864.html) |  |  |
| 93F11--14;94D10--13 | Df(3R)[hh](http://flybase.org/reports/FBab0002795.html) |  |  |
| 94D2--10;94E1--6 | Df(3R)BSC55 |  |  |
| 95A5--7;95D6--11 | Df(3R)mbc-R1 |  |  |
| 95F6-8;96A18-20 | Df(3R)crb87-5 |  |  |
| 96A2--7;96D2--4 | Df(3R)slo8 |  |  |
| 96F1;97B1 | Df(3R)Espl3 |  |  |
| 96F12--14;97C4--5 | Df(3R)ME61 |  |  |
| 97A;98A1--2 | Df(3R)[Tl-P](http://flybase.org/reports/FBab0002666.html) |  |  |
| 98D3--7;98D3--7 | Df(3R)[M15](http://flybase.org/reports/FBab0027162.html) |  |  |
| 98E3;99A6--8 | Df(3R)[3450](http://flybase.org/reports/FBab0022382.html) |  |  |
| 99A6;99C1 | Df(3R)01215 |  |  |
| 99B5--6;99F1 | Df(3R)L127 |  |  |
| 99F1--2;100B4--5 | Df(3R)tll-g | Whiter eyes |  |
| 100A2;100C2--3 | Df(3R)tll-e |  |  |
| 100D2;100F5 | Df(3R)04661 |  |  |

**Table S1**

**Results of Deficiency screen**. Six mosaic *roX1* lines were assayed in the deficiency screen (Bloomington Deficiency collection) and the deficiencies that dominantly suppressed the red pigmentation across more than four different mosaic *roX* lines are indicated. Candidate genes that have been mapped within those deficiencies are also shown. Only ten intervals (14/ 190 deficiencies) assayed had an effect on the eye phenotype indicating that a general suppression of transcription does not lower the expression of the dosage compensation reporter. *Spt5* would be an eleventh locus, but is not uncovered in the Df collection.
