# Supplementary material for: Mutations in the Transcription Elongation Factor SPT5 Disrupt a Reporter for Dosage Compensation in Drosophila
Source: PLoS Genet. 2012 Nov 29;8(11):e1003073. doi: 10.1371/journal.pgen.1003073 (PMC3510053; doi:10.1371/journal.pgen.1003073)
Supplement: Table S2 — Lowering Spt5 decreases male viability. In the absence of both roX1 and roX2 males are dead. The male specific lethality can be rescued by an autosomal GMroX1 transgene. In this assay roX1-,roX2-; [GMroX1-75C] virgins were crossed to balanced males carrying the indicated mutations. The male progeny of this cross that are roX1-,roX2-/Y;mutation/+;[GMroX1-75C]/+ are compared to the nonbalanced female progeny. All the flies therefore carry only one copy of the indicated mutation. The statistical significance was determined by Fisher exact fit test. Figure 2A. Graphical representation of this analysis. (DOC) [file pgen.1003073.s008.doc]

**Table S2**

| Genotype | Males | Females | %male viability |
| --- | --- | --- | --- |
| Wild type | 167 | 573 | 29.1 |
| *msl1^L60^* | 0 | 365 | 0 |
| *mle* | 71 | 422 | 16.8 |
| *Spt5^Q314X^* | 49 | 321 | 15.2 |
| *Spt5^E471Z^* | 61 | 302 | 20.2 |
| *Spt5^A680Z^* | 127 | 630 | 20.2 |
| *Su(Tpl)^S192^* | 137 | 750 | 18.3 |
| *Jil1^z2^* | 107 | 356 | 30.0 |
| *Elongin-C^G0635^* | 225 | 753 | 29.9 |

**Lowering *Spt5* decreases male viability**. In the absence of both *roX1* and *roX2* males are dead. The male specific lethality can be rescued by an autosomal *GMroX1* transgene. In this assay *roX1^-^,roX2^-^; [GMroX1-75C]* virgins were crossed to balanced males carrying the indicated mutations. The male progeny of this cross that are *roX1^-^,roX2^-^/Y;mutation/+;[GMroX1-75C]/+* are compared to the nonbalanced female progeny. All the flies therefore carry only one copy of the indicated mutation. The statistical significance was determined by Fisher exact fit test. Figure 2A. Graphical representation of this analysis
