# Supplementary material for: Mutations in the Transcription Elongation Factor SPT5 Disrupt a Reporter for Dosage Compensation in Drosophila
Source: PLoS Genet. 2012 Nov 29;8(11):e1003073. doi: 10.1371/journal.pgen.1003073 (PMC3510053; doi:10.1371/journal.pgen.1003073)
Supplement: Text S1 — Supplementary materials and methods. (DOC) [file pgen.1003073.s009.doc]

**Supplementary Materials and Methods**

**Mutagenesis**

Males isogenic for the second chromosome were fed 25 mM EMS overnight, rested 1 day

and then mated to *iso2* virgins. The F1 male and female progeny which carry

mutagenized chromosomes were crossed to the opposite sex of either *GMroX1-60F* or

*GMroX1-69C* flies. Candidate males recovered showed a dramatic lowering of mosaic

pigmentation. The design of the screen prevented recovery of X-linked mutations.

**Complementation tests**

Balanced males from each of the modifier mutants were mated to each other or to the

deficiencies and the presence of nonbalanced progeny was scored.

**Antibody generation**

Plasmids for bacterial expression of SPT5 protein fragments, SPT5 fragments SPT5N (aa

112-393), SPT5M (aa 389-733) and SPT5C (aa 732-1054) expressed as MBP fusion

proteins were a kind gift from Dr. John Lis [21]. Fusion proteins were

induced with 0.25mM IPTG at 30-32^○^C for 2 h. Bacterial pellet was resuspended in the

lysis buffer AB1 (20 mM HEPES pH7.9, 200 mM KCl, 1 mM EDTA with 1 mM PMSF)

with lysozyme and lysed by sonication. Lysates were cleared and bound to equilibrated

amylose beads and allowed to bind the lysate for 2 h at 4^○^C. After washes with 100 ml of

lysis buffer, the proteins were eluted with AB2 elution buffer (20 mM HEPES pH7.9, 20

mM KCl, 1 mM EDTA with 1 mM PMSF ) with 20 mM maltose. Fusion protein purity

was checked by Coomassie staining and concentration was estimated by Bradford’s

reagent (BioRad) according to manufacturer protocol. 1mg of a mixture of protein

fragments was used to immunize guinea pigs and antibodies were generated by Cocalico

Biological, Pennsylvania.

**Polytene squashes**

Polytene squashes were prepared as described in [58] with the following

changes. For anti-RNAPII and anti-H3K36me3 staining, 2%

nonylphenoxylpolyethoxylethanol (NP40) was included in addition to 1% Triton X-100

in fix I. Primary antibodies were rabbit anti-MSL1 antibodies (1:50 dilution), guinea pig

anti-SPT5 antibodies (1:100), mouse H5 monoclonal anti-Ser2P RNAP (Covance, 1:30),

mouse H14 monoclonal anti-Ser5P RNAP (Covance, 1:50) and rabbit anti-H3K36me3

(Invitrogen, 1:50). Secondary antibodies were used in combinations that allowed for dual

protein localization and include anti-Rabbit Cy2 (Jackson Immunoogical 1:500 dilution),

anti-Rabbit Texas Red (Jackson Immunological) or anti-Rabbit AF488 (Invitrogen), anti-

Guinea pig Texas Red (Jackson Immunological), for detecting H5 and H14 primary anti-

Mouse IgM Cy3.5 (Jackson Immunological) and for H14 primary anti-mouse AF488

(Invitrogen) at 1:200.

**Chromatin Immunoprecipitation**

S2 cells at a concentration of 2-3X 10^6^ cells/ml were crosslinked with 1% formaldehyde

for 10 min at room temperature. To stop crosslinking glycine was added to a final

concentration of 0.25mM and incubated for 5 min at room temperature. Cells were

collected, washed twice with ice cold 1X PBS-EDTA and resuspended in nuclear

extraction buffer (15 mM HEPES, 5 mM MgCl2, 0.2 mM EDTA, 0.5 mM EGTA, 10

mM KCl, 350 mM Sucrose, 0.1% Tween 20, 0.5 mM PMSF, 1 mM DTT with protease

inhibitor). The cells were disrupted in a dounce homogenizer. The nuclei were collected

in Pre RIPA (10 mM Tris, 0.1% SDS and 0.1 mM EDTA). To obtain fragmented

chromatin, 300 μl of Chromatin was sonicated in a Diagenode bioruptor in a 30 sec

ON/OFF cycle at Hi power setting. Chromatin was sheared to 300-700 bp fragments for

immunoprecipitation. Sheared chromatin was made upto 1% TritonX-100, 0.1%sodium

deoxycholate, 150 mM NaCl and protease inhibitors.

Chromatin was precleared for 1 h at 4^○^C with preblocked Protein A beads. 10% of

chromatin fraction was removed for input control. For anti-MSL1 pulldowns, 1 μl of anti-

MSL1 ChIP grade antibodies (gift from M.Kuroda) was incubated with chromatin

overnight at 4^○^C.with rotation. Immune complexes were harvested by rotating with 50%

preblocked. Protein A bead slurry at 4^○^C for 2.5 h. All washes were performed for 10 min

at 4^○^C Washes were as follows. Two washes with RIPA 150 (50 mM Tris HCl, 1%

NP40, 2 mM EDTA 150 mM NaCl, 0.1% SDS, 0.5 mM PMSF, 1 mM DTT),one wash

with RIPA 300 (50 mM Tris HCl, 1% NP40, 2 mM EDTA 300 mM NaCl, 0.1% SDS, 0.5

mM PMSF, 1 mM DTT) one wash with LiCl Immune complex wash buffer (100 mM

Tris-HCl, 1% NP-40, 2 mM EDTA, 250 mM LiCl, 1% DOC) and finally two washes

with TE (10 mM Tris pH 7.6; 1 mM EDTA) . The bound chromatin was eluted with 250

μl of ChIP elution buffer (1% SDS and 50 mM Sodium bicarbonate) by rotating 20 min

at room temperature two times. Eluted DNA was reverse crosslinked at 65^○^C overnight

and purified by phenol-chloroform extraction. Final pellet was resuspended in 50 μl of

water and 1μl was used for PCR reactions.

The following primers were used for PCR

| Primer | Sequence |
| --- | --- |
| roX2DHS F | CTT TCG TTT AGG TAG CTC GGA TG |
| roX2DHS R | ACT ATG CGG AAA TCG TTA CTC TTG CT |
| PKA F | AGG TAG CCC TGC GAG TCA A |
| PKA R | GCT TCT ACG CGG CGC AAA T |
| CG13316 5’F | TAGTTTGCTTCTGTCATTGCATTCGTCG |
| CG13316 5’R | GGTGACGGATTCGGATTCTTGTGT |
| CG13316 3’F | AGAAGCGGCTAAAGCAACTGAG |
| CG13316 3’R | AGATGCGAGCAAATGGATGGAG |
| CG32767 5’F | TTGTTTGCGGGGTGTGTGC |
| CG32767 5’R | AGCATCAGGCTTTGGTCACATTAC |
| CG32767 3’F | CGGATATAGCTGCACAGGTATGCT |
| CG32767 3’R | CCAAGCTCGAAATTCCGTATCTCCA |

**Protein interaction**

MBP-SPT5N, MBP-SPT5M, MBP-SPT5C as well as the unrelated protein MBP-MCP

were induced in bacteria with 0.025 mM IPTG for 2 h. Additionally GST-MSL1 C-terminal

domain fusion protein and GST were also induced with 0.025 mM IPTG for 2 h.

Cells were pelleted and lysed with either MBP-lysis buffer for MBP fusion proteins (20

mM Tris pH7.5, 200 mM NaCl, 1 mM EDTA, 10 mM β-mecaptoethanol along with

PMSF (phenyl methyl sulfonyl fluoride), protease inhibitors and lysozyme) or GST lysis

buffer (50 mM Tris pH 7.5, 500 mM NaCl, 0.5% NP40, 5 mM EDTA, 5 mM EGTA

along with PMSF, protease inhibitors and lysozyme) for GST proteins. Lysates were

clarified and bound either to amylose beads equilibrated in MBP lysis buffer to purify

MBP fusion proteins or glutathione beads equilibrated in GST lysis buffer to purify GST

fusion proteins. For purifying MBP fusion proteins, beads were washed thrice with MBP

lysis buffer and then beads were stored as a 50% slurry in MBP lysis buffer at 4^○^C till the

setting up of binding. For purifying GST fusion proteins, beads were washed three times

with GST lysis buffer, two times with TEE (200 mM Tris (pH 8.0, 5 mM EDTA and 5

mM EGTA) and then eluted with 50 mM glutathione in TEE.

Concentrations of the proteins was evaluated by Bradford’s assay and purity of protein

preparations was visualized by Coomassie blue staining of SDS- polyacrylamide gel

analysis of the purified proteins. To set up the binding, equivalent molar concentrations

of the proteins were used. MBP proteins bound to amylose beads were equilibrated in

binding buffer (MBP lysis buffer with 5 mg/ml BSA, 0.5% NP40 and 1 mM DTT) for 1 h

at 4^○^C and eluted GST proteins were diluted ten-fold in binding buffer and allowed to

interact with the beads at 4^○^C overnight. Beads were washed three times with wash buffer

(20 mM Tris , 150 mM NaCl and 0.1% NP40) for 10 mins with rotation at 4^○^C. Bound

proteins were eluted by boiling in SDS-loading buffer. Proteins were separated on 8%

SDS-polyacrylamide gels and Westerns were performed as detailed (Prabhakaran and

Kelley, 2010). For detecting GST, anti-GST antibodies (Sigma, 1:750 dilution) and HRP

conjugated anti-mouse secondary antibodies (Jackson Immuno, 1:5,000) dilution was

used. For detecting MBP-bound SPT5, affinity purified guinea pig anti-SPT5 sera

(1:1,000) and anti-guinea pig HRP conjugated antibodies (Jackson Immuno, 1:5,000) was

used. The proteins were visualized by lunimol reagent (Santa Cruz). For inputs, films

were exposed for 20 seconds and for detecting pulldowns, films were exposed for 30

seconds
